# *scPred*: accurate supervised method for cell-type classification from single-cell RNA-seq data

**DOI:** 10.1186/s13059-019-1862-5

**Published:** 2019-12-12

**Authors:** Jose Alquicira-Hernandez, Anuja Sathe, Hanlee P. Ji, Quan Nguyen, Joseph E. Powell

**Affiliations:** 1 Garvan Institute of Medical Research, Darlinghurst, Sydney, Australia; 2 Institute for Molecular Bioscience, University of Queensland, Brisbane, Australia; 3 Division of Oncology, Department of Medicine, Stanford University School of Medicine, Stanford, USA; 4 Stanford Genome Technology Center, Stanford University, Palo Alto, USA; 5 Faculty of Medicine, University of New South Wales, Darlinghurst, Sydney, Australia

## Abstract

Single-cell RNA sequencing has enabled the characterization of highly specific cell types in many tissues, as well as both primary and stem cell-derived cell lines. An important facet of these studies is the ability to identify the transcriptional signatures that define a cell type or state. In theory, this information can be used to classify an individual cell based on its transcriptional profile. Here, we present *scPred*, a new generalizable method that is able to provide highly accurate classification of single cells, using a combination of unbiased feature selection from a reduced-dimension space, and machine-learning probability-based prediction method. We apply *scPred* to scRNA-seq data from pancreatic tissue, mononuclear cells, colorectal tumor biopsies, and circulating dendritic cells and show that *scPred* is able to classify individual cells with high accuracy. The generalized method is available at https://github.com/powellgenomicslab/scPred/.

## Introduction

**Fig. 1:**
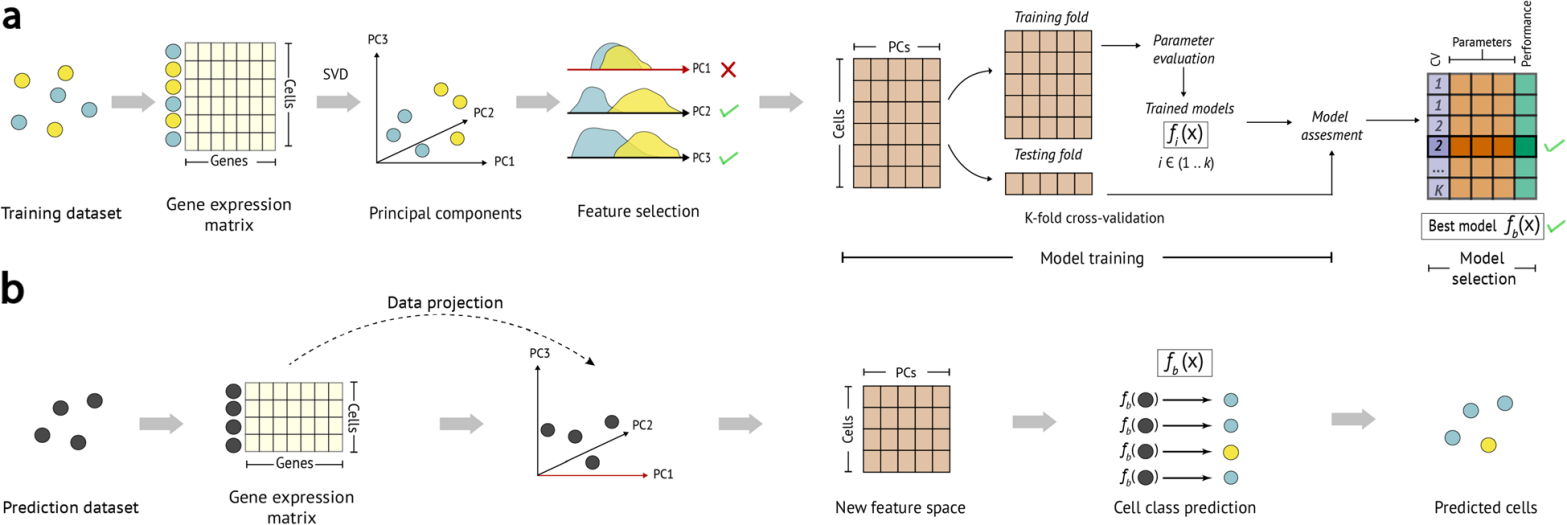
Summary of the scPred method. **a** Training step. A gene expression matrix is eigendecomposed via singular value decomposition (SVD) to obtain orthonormal linear combinations of the gene expression values. Only PCs explaining greater than 0.01% of the variance of the dataset are considered for the feature selection and model training steps. Informative PCs are selected using a two-tailed Wilcoxon signed-rank test for each cell class distribution (see the “Methods” section). The cells-PCs matrix is randomly split into k groups and the first *k* group is considered as a testing dataset for cross-validation. The remaining *K*-1 groups (shown as a single training fold) are used to train a machine learning classification model (a support vector machine). The model parameters are tuned, and each *k* group is used as a testing dataset to evaluate the prediction performance of a *f*_i_(*x*) model trained with the remaining *K*-1 groups. The best model in terms of prediction performance is selected. **b** Prediction step. The gene expression values of the cells from an independent test or validation dataset are projected onto the principal component basis from the training model, and the informative PCs are used to predict the class probabilities of each cell using the trained prediction model(s) *f*_b_(*x*)

Individual cells are the basic building blocks of organisms, and while a human consists of an estimated 30 trillion cells, each one of them is unique at a transcriptional level. Performing bulk or whole-tissue RNA sequencing, which combines the contents of millions of cells, masks most of the differences between cells as the resulting data comprises of the averaged signal from all cells. Single-cell RNA-sequencing (scRNA-seq) has emerged as a revolutionary technique, which can be used to identify the unique transcriptomic profile of each cell. Using this information, we are now able to address questions that previously could not be answered, including the identification of new cell types [1–4], resolving the cellular dynamics of developmental processes [5–8], and identify gene regulatory mechanisms that vary between cell subtypes [9]. Cell type identification and discovery of subtypes has emerged as one of the most important early applications of scRNA-seq [10]. Prior to the arrival of scRNA-seq, the traditional methods to classify cells were based on microscopy, histology, and pathological criteria [11]. In the field of immunology, cell surface markers have been widely used to distinguish cell subtypes [12], for a wide range of purposes. While this approach is desirable in practical terms for cell isolation, e.g., via fluorescence-activated cell sorting (FACS), these markers may not reflect the overall heterogeneity at a transcriptomic and phenotypic level from mixed cell populations [13, 14]. Unsupervised and supervised clustering approaches have been used to determine groups of cells based on similar transcriptional signatures within a sample [2, 15, 16], and frequently, cells within a cluster are collectively labeled based on the average expression levels of canonical markers [17]. The cluster-based classification methods assume that all cells within a cluster are the same type and thus can be labeled collectively. This assumption is frequently wrong, with clusters often containing small percentages of multiple cell types in addition to a major cell type. A method that classifies each cell individually, without clustering first, solves these problems and should provide higher overall accuracy in cell labeling. To be able to predict the classification of a single cell based upon its transcriptome read-out, first, a prediction model needs to be built where the effects of given features are estimated. It is clear that both the selection of features and estimation of their effects play a critical role in the overall prediction performance. Unlike prediction methods that use data derived from bulk RNA-seq data where gene expression averages are used as features, phenotype prediction at single-cell level faces new challenges. Firstly, cell-to-cell differences must be considered to define and predict cell types. Using only a subset of genes (e.g., differentially expressed genes) will likely exclude discriminant sources of variation across cells. An additional limitation is the inconsistency seen between statistical methods used to identify differentially expressed genes [18]. Finally, if the number of observations that define a specific subtype of cells is high, then classification algorithms can be computationally expensive or suffer from overfitting. There are numerous applications for which prediction of a cell state or type from its scRNA-seq data can play an important role. An obvious example is in the burgeoning use of single-cell data in characterizing disease states and underlying biology at single-cell resolution [12, 19]. The granular nature of single-cell characterization has enormous implications for the accurate prediction of specific cell subtypes and pathological-related states. We anticipate that such prediction strategies will play an important role in the early diagnosis of diseases or informing personalized treatment. Similarly, efforts arising from the Human Cell Atlas [10] are set to create a comprehensive reference atlas of most cell subtypes in the human body, meaning cells from new samples can be mapped against this reference. Here, we introduce *scPred*, a method that takes advantage of dimensionality reduction and orthogonalization of gene expression values to accurately predict specific cell types or states of single cells from their transcriptional data (see Fig. 1). *scPred* can be applied to any situation where cells can be labeled into discrete categories, including cell subtypes or defined cell states.

## Results

*scPred* is a generalized method for classifying a single cell based on its transcriptional data. The method uses a combination of decomposing the variance structure of a gene expression matrix to identify limited informative features, and a machine learning approach to estimate the effect of these features on classifying cells (Fig. 1). In doing so, it is able to incorporate a large number of small differences in the mean and variance of gene expression between different cell types in the prediction model. This removes the need to perform gene-specific analyses such as to identify informative features. *scPred* has two main steps. Firstly, a prediction model is built using a training cohort of single-cell data, where the identity of the cells is already known. Secondly, the application of the prediction model to single-cell data obtained from independent sample, with each cell then assigned a conditional class probability (*y = 1|f*) of belonging to a given cell subtype or state. *scPred* incorporates a rejection option to avoid assigning cells to a class when the conditional class probability is lower than a given value. In the case of binary classification, this means that Pr*(y = 1|f)* and Pr*(y = 0|f)* should be higher than a probability threshold for a cell to be assigned to any class respectively. When the maximum probability across all classes is lower than the threshold, then a cell is labeled as “unassigned.” Sensitivity and specificity metrics reported in this paper consider the “unassigned” label as incorrect. Therefore, computed probabilities for cells in the test dataset are below the threshold, all of them are labeled as unassigned, and the sensitivity and specificity are both equal to zero (see the “Methods” section). This criterion avoids misclassifying cells when some cell types are not considered in the prediction model but are present in the test data. For all analyses in this paper, we used a strict threshold of 0.9. This threshold can be changed in the software implementation. Here, we present the results of the application of *scPred* under four distinct scenarios.

### *scPred* can accurately predict tumor epithelial cells from gastric cancer

**Fig. 2:**
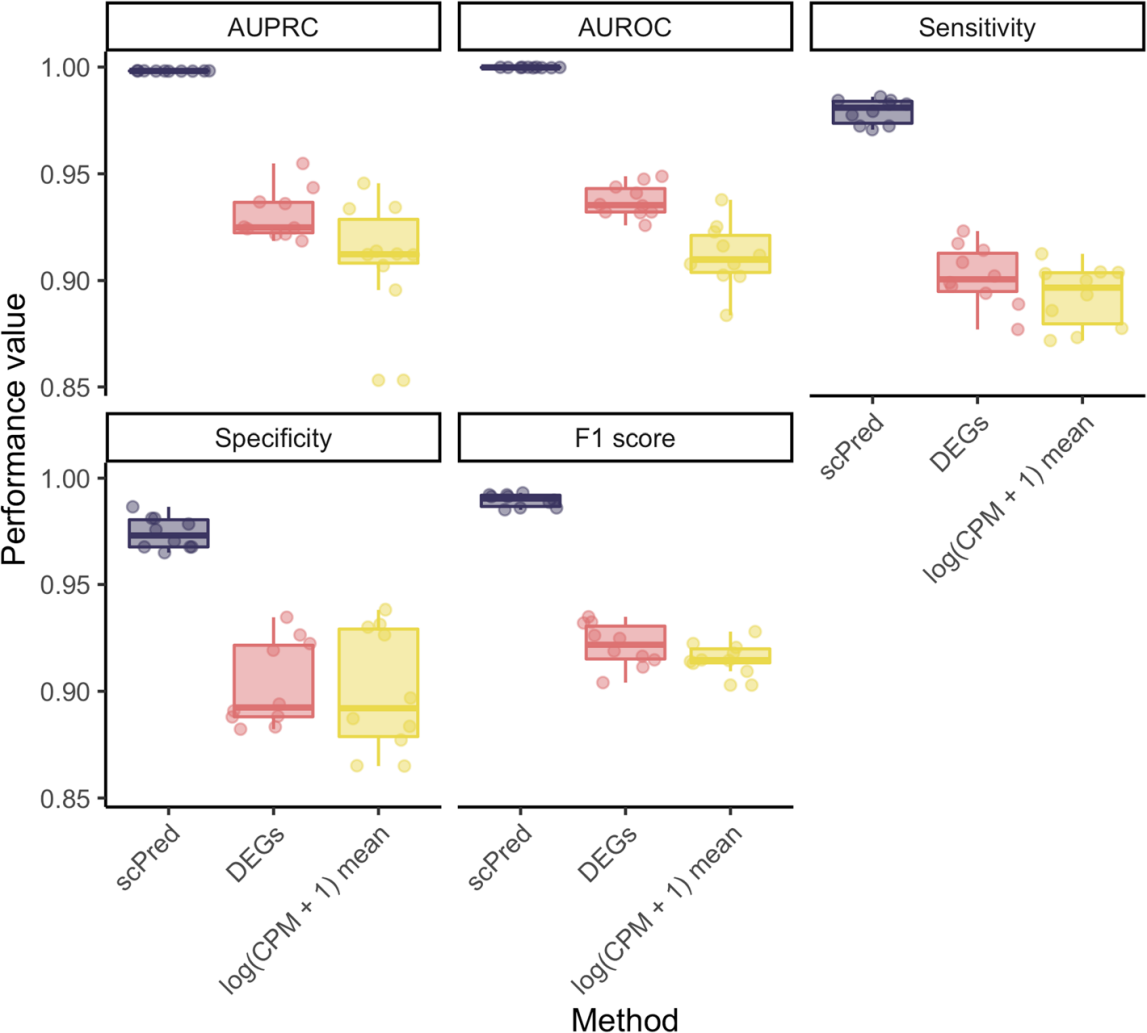
Classification performance of tumor cells from gastric adenocarcinoma. scPred shows high prediction accuracy to classify tumor cells (0.979 (95% bootstrap CI 0.973–0.984) and non-tumor cells 0.974 (95% bootstrap CI 0.960–0.989). scPred outperforms predictions based on differentially expressed genes and per-cell mean of log2(CPM + 1) (prediction baseline). Ten bootstrap replicates were used to assess the prediction performance of all methods

We initially sought to demonstrate the performance of *scPred* by making computational predictions of tumor and non-tumor cells based on their transcriptomes and validating our classification using a cell-specific independent immunohistochemistry assay targeting the MLH1 and PMS2 protein expression. We used this information as an RNA-independent gold-standard to annotate the status of all cells. The loss of MLH1 protein expression has been shown to be related to a hypermutable state of microsatellite instability which can be detected at single-cell resolution from the raw scRNA-seq data [20]. Along with the protein depletion confirmed by the immunochemistry assay, we confirmed the loss of expression of MLH1 at the RNA level, as well as the upregulation of EPCAM and TFF3 in tumor cells, which are known to be overexpressed in cancer cells [21, 22] (see Additional file 1: Figure S1). Thus, we have a method for linking the observed scRNA-seq data to the immunohistochemistry-labeled classification for each cell. We obtained surgical biopsies from stage IIA intestinal gastric adenocarcinoma along with matched-normal epithelium from two patients and measure the protein expression of MLH1 and PMS2 to validate the presence of tumor cells. Then, we generated scRNA-seq data for four samples using the Chromium platform (10X Genomics). For the first pair of samples, we obtained 1905 epithelial cells based on the expression of EpCAM, and from these identified tumor cells based on the microsatellite stability, and trained a model to detect differences between tumor and non-tumor epithelial cells. Using cells from the second pair of samples, we then applied this model to classify cells using just the scRNA-seq data. We then confirmed our predictions using the observation of microsatellite instability. Overall, we obtained a sensitivity of 0.979 and specificity of 0.974 (AUROC = 0.999, AUPRC = 0.999, F1 score = 0.990) across the ten bootstrap replicates (Fig. 2 and Additional file 2: Table S1). To demonstrate the increase in performance from using the principal components selected by *scPred* as features, we compared the prediction performance of *scPred* against five baselines. First, we fitted an SVM model using *scPred* and used only the intercept of the hyperplane as a predictor to evaluate any bias in the predictor due to class proportions. We obtained sensitivity and specificity equal to zero (AUROC = 0.5 and AUPRC = 0) and thus show no bias when cell classification is attempted for unequal ratios of cells. Then, we evaluated the contribution of the informative principal components by setting all the coefficients of the hyperplane to 1. We found that the sensitivity was 0 and the specificity 0.995 (AUROC = 0.496, AUPRC = 0.538, F1 = 0.000), which indicates that the predictors should be weighted to separate the cells accurately into tumor and non-tumor. To demonstrate the importance of the feature selection performed by *scPred*, we used all principal components as predictors to having a baseline of the expected classification from all data. We obtained sensitivity and specificity equal to zero (AUROC = 0 and AUPRC = 0.398, F1 = 000), which implies that the informative principal components recover the cell-type informative variance from the entire data. To evaluate how the global cell composition can predict tumor status, we calculated the per-cell mean of the log2(CPM + 1) and trained a model using these values as a predictor. After performing the predictions on the test data, we obtained a sensitivity of 0.894 and specificity of 0.902 (AUROC = 0.912, AUPRC = 0.912, F1 = 0.916). The lower performance compared to the default *scPred* algorithm demonstrates using principal components for feature selection captures variance in gene expression that cell-type specific. Finally, we calculated the differentially expressed genes between tumor and non-tumor cells to obtain a set of discriminant genes. We used these genes as features to train a model, and after the predictions, we obtained sensitivity and specificity of 0.903 and 0.909 respectively (AUROC = 0.937, AUPRC = 0.931, F1 = 0.922). For all analyses, we performed ten bootstrap replicates with the same data partitions used before. These results show that *scPred* yields higher accuracy than our baseline models and outperforms predictions based on differentially expressed genes (see Fig. 2 and Additional file 2: Table S1). Next, we investigated the effect of sequencing depth and the number of cells of a given cell type on the prediction performance of *scPred*. First, we down-sampled the reads from each cell in the training data by scaling the gene counts so that each cell had fewer than a fixed *N* number of reads. We evaluated a range of sequencing depths from an average of 5000 to 40,000 reads per cell to train the models and predicted the cell types from the test data. We repeated this process ten times using the same data partitions from previous analyses. The sensitivity of the classification showed no changes across sequencing depths, while the specificity, AUROC, and AUPRC showed a considerable decrease once the average reads per cell are 20,000 (Additional file 1: Figure S2). Based on the cellranger output, we estimate that 20,000 reads per cell represent approximately 50% saturation of the library. We, therefore, recommend training models on cells that have been sequenced to high saturation. Finally, we assessed the impact of the cell population size in the prediction accuracy by performing bootstrap iterations of classifying cells using a training model generated with between 100 and 900 randomly sampled tumor cells. We observed a small effect on the AUROC, AUPRC, F1 score, sensitivity, and specificity until the number of tumor cells included in the training model was less than 200. When only 100 cells were included (AUROC = 0.996, AUPRC = 0.996, F1 = 0.990), the mean sensitivity dropped down to 0.741 while the specificity changed from 0.974 to 0.885 with respect to the 953 cells used originally (see Additional file 1: Figure S3). Collectively, these results show that *scPred* can accurately classify cells provided they are not a very rare type in the training data.

### *scPred* can accurately predict cell subtype using scRNA-seq data generated across different platforms

**Fig. 3:**
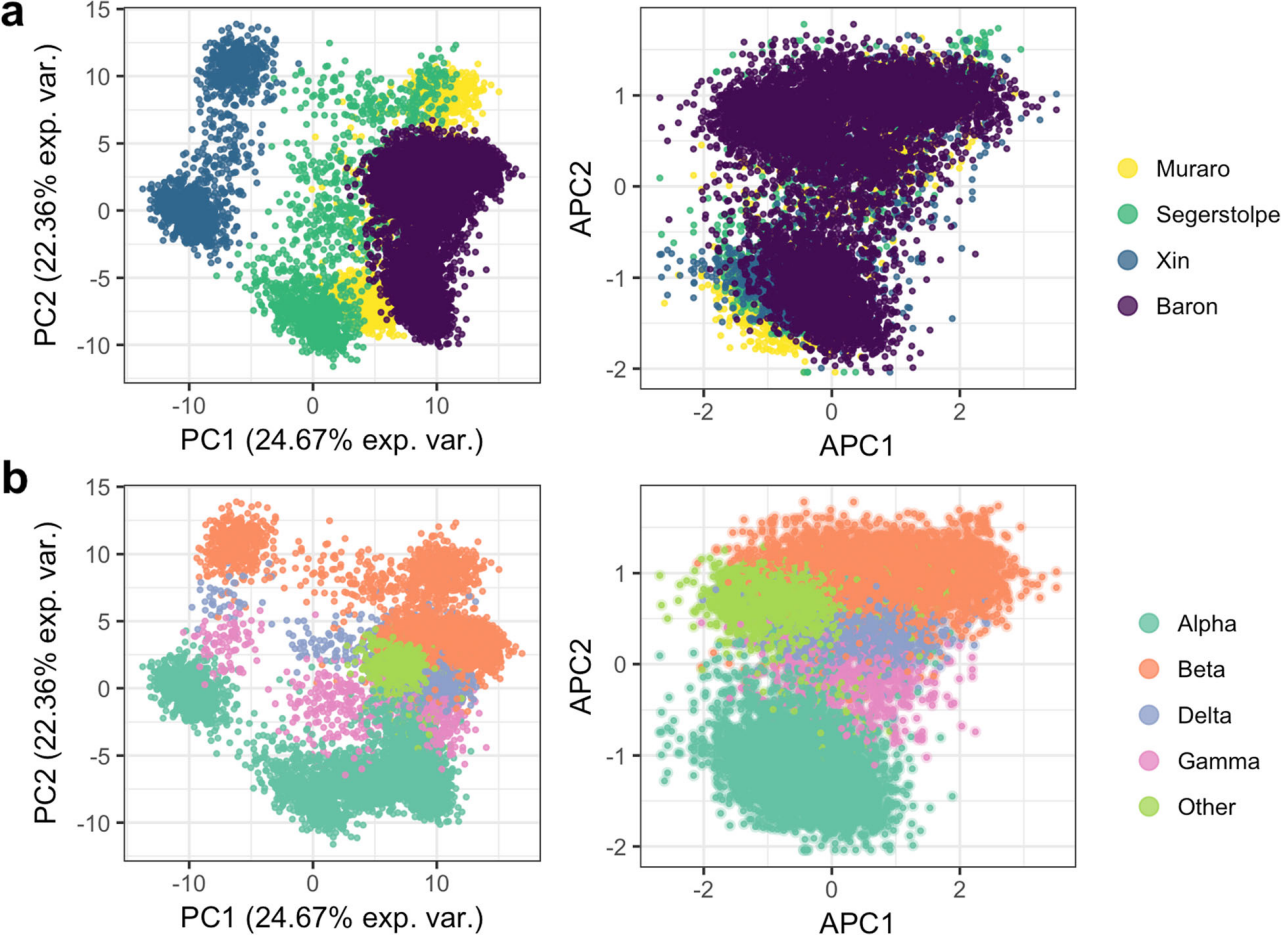
Principal component alignment of pancreatic cells. **a** Training (Muraro, Segerstolpe, and Xin) datasets [3, 25, 26] were used to generate the training eigenspace. The test dataset (Baron et al. [31]) was projected, and all datasets were aligned using Seurat. No batch effect is observed after the alignment. **b** α, β, δ, and γ cells are included in the training datasets. The prediction dataset contains also 2326 “other” cell types such as epsilon, acinar, stellate, ductal, endothelial, Schwann, and T cells (bright green cells). After the dataset alignment, cells cluster by cell type. The *X*-axis shows variance explained (exp.var.), principal components (PC), and aligned principal components (APC)

Given the rapid development of single-cell sequencing assays and technologies, we anticipate that a prediction model for a given cell subtype(s) will often be built with data generated from an alternative platform to that used for independent test samples. To assess the robustness of *scPred*, we sought to evaluate the performance using training data generated from multiple platforms and testing the prediction accuracy for independent cells sequenced on another platform. We chose to develop a prediction model using *scPred* to classify subtypes of islets of Langerhans cells from scRNA-seq data generated from pancreas tissue due to their limited abundance (4.5% in a pancreatic tissue sample) [23], and thus will represent a class of cells that is expected to be more difficult to predict based on their low relative existence compared to other cells. Islets of Langerhans are composed mainly of four distinct cell types, namely α (alpha), β (beta), δ (delta), and γ (gamma) cells, that are responsible for producing glucagon, insulin, somatostatin, and pancreatic-polypeptides, respectively [24]. We generated a training reference cohort of scRNA-seq data from a total of 4292 cells from three independent studies undertaken by Muraro et al. [25], Segerstolpe et al. [3], and Xin et al. [26] that had sequenced cells using CEL-seq2 [27], Smart-Seq2 [28], and SMARTer [29], respectively. Details of the training cohort data are given in Additional file 2: Table S2. Importantly, using the Seurat alignment method [30], we are able to demonstrate that between platform and between sample batch effects can be removed for the training cohort (Fig. 3). The best fit models from *scPred* for α, β, δ, and γ cells used between 14 and 18 PCs, which represents a small feature space for prediction in an independent data, and correspondingly will reduce the computational requirements of *scPred* in the testing phase. Using the prediction classifier model trained from the aligned reference cohort data, we naively predicted the cell type of each of 7932 cells [31], collected from four healthy individuals, using their scRNA-seq data generated using inDrop [32]. The testing data includes a heterogeneous mix cell islets of Langerhans cells, meaning non-α, β, δ, and γ cells, such as epsilon, endothelial, or T cells, provide a negative control. We classified a cell as a specific cell subtype based on a class probability (Pr*(y = 1|f)*) greater than 0.9. The overall accuracy of the predictions was evaluated based on the known cell identities determined based on the expression of classic markers (GCG, INS, SST, and PPY). For islets of Langerhans cells, the prediction model built by *scPred* using the scRNA-seq data from the reference cohort was able to predict cell type with an average accuracy of 97.68% (Table 1 and Fig. 3) and accurately labeling 94.9% heterogeneous populations of other cells. For example, of the 2302 α cells in the test cohort, our *scPred* model classified 2264 cells correctly. Of the 38 misclassified cells, 33 were unassigned to another target cell type, which also demonstrates a high specificity of the model. We observed the same pattern for all cell types tested (Additional file 2: Table S3). To further support this conclusion, the mean Pr*(y = 1|f)* for cells classified as α, β, δ, and γ was 0.994–0.997, while cells classified as other (i.e., epsilon, endothelial, or T cells) had a mean Pr*(y = 1|f)* of 0.307 (Additional file 1: Figure S4). To evaluate the effect of the Seurat manifold alignment on data integration across platforms, we performed the sample cell classifications using trained models developed using unaligned data. We observed a reduction of 31% in sensitivity and only 0.7% in specificity (see Additional file 3: Table S4). Then, we compared the performance of other prediction models (k-nearest neighbors, elastic net, Naive Bayes, multivariate adaptive regression splines, random forests, and generalized linear model) to support vector machines. Overall, support vector machines with a radial kernel showed the highest accuracy for detecting cells from the islets of Langerhans while reducing the proportion of other cells being miss-classified (see Additional file 2: Table S5). Together, these results show that support vector machines are ideal for classifying single cells from an informative feature eigenspace.

**Table 1:**
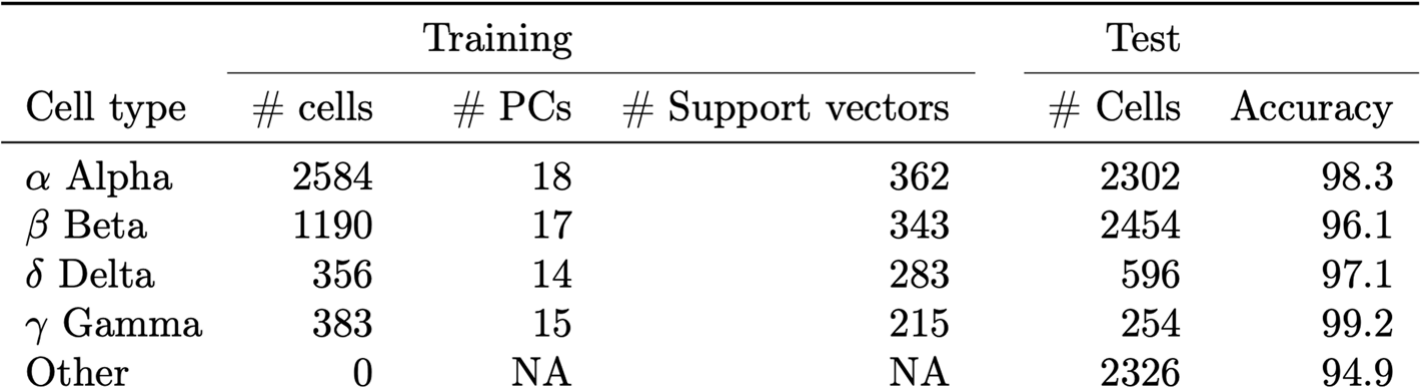
Prediction of pancreatic cells. The training panel corresponds to the Muraro, Segerstolpe, and Xin datasets used as a reference to train the prediction models for each cell type from the islets of Langerhans. As part of the training, no other cell types were considered. The test information corresponds to the Baron [31] dataset used to measure the performance of the trained models in an independent dataset. The Baron dataset contains epsilon, acinar, stellate, ductal, endothelial, Schwann, and T cells referred as “Other” in this table. The accuracy is defined as the fraction of cells correctly assigned for each cell type of interest. The accuracy for the remaining cells corresponds to the fraction of cells from the test dataset that are correctly unassigned to any of the classes of interest (negative controls) as a consensus across all four prediction models

To further demonstrate the application of a *scPred* model in multiple datasets, we trained a prediction model on the Baron data and assessed its prediction performance on the Muraro, Segerstolpe and Xin datasets. We obtained an average accuracy of 0.98, 0.92, 0.93, and 0.82 for alpha, beta, delta, and gamma cells respectively using the Baron dataset as reference only (see Additional file 2: Table S6). Finally, we compared the performance of *scPred* with *scmap* [33], *CaSTLe* [34], *singleCellNet* [35], and *scID* [36] methods. For *scmap*, we applied both cluster and cell projection algorithms to annotate cells based on their proximity to the reference dataset. *Scmap* failed to identify all gamma cells using both algorithms and exhibited low performance classifying delta cells by yielding at most 14% of accuracy using the Baron dataset as reference (see Additional file 2: Tables S7 and S8). Likewise, *CaSTLe* exhibited an accuracy of zero for classifying gamma cells in both Segerstolpe and Xin datasets and very poor accuracy to detect beta and delta cells (see Additional file 3: Table S9). *singleCellMap* suffered from very low accuracy for gamma cells with only 9% of accuracy (see Additional file 3: Table S10). *scID* classified gamma cells from the Segerstolpe and Xin datasets with high accuracy (96% and 94% respectively), however, it failed to classify 99% from the Muraro dataset (see Additional file 3: Table S11). These results show that the features selected from the decomposed training data are able to define hyperplanes that are able to separate individual cells by cell type, based upon linear combinations of scRNA-seq data fitted to a *scPred* models.

### Accurate prediction of peripheral blood mononuclear cells

**Fig. 4:**
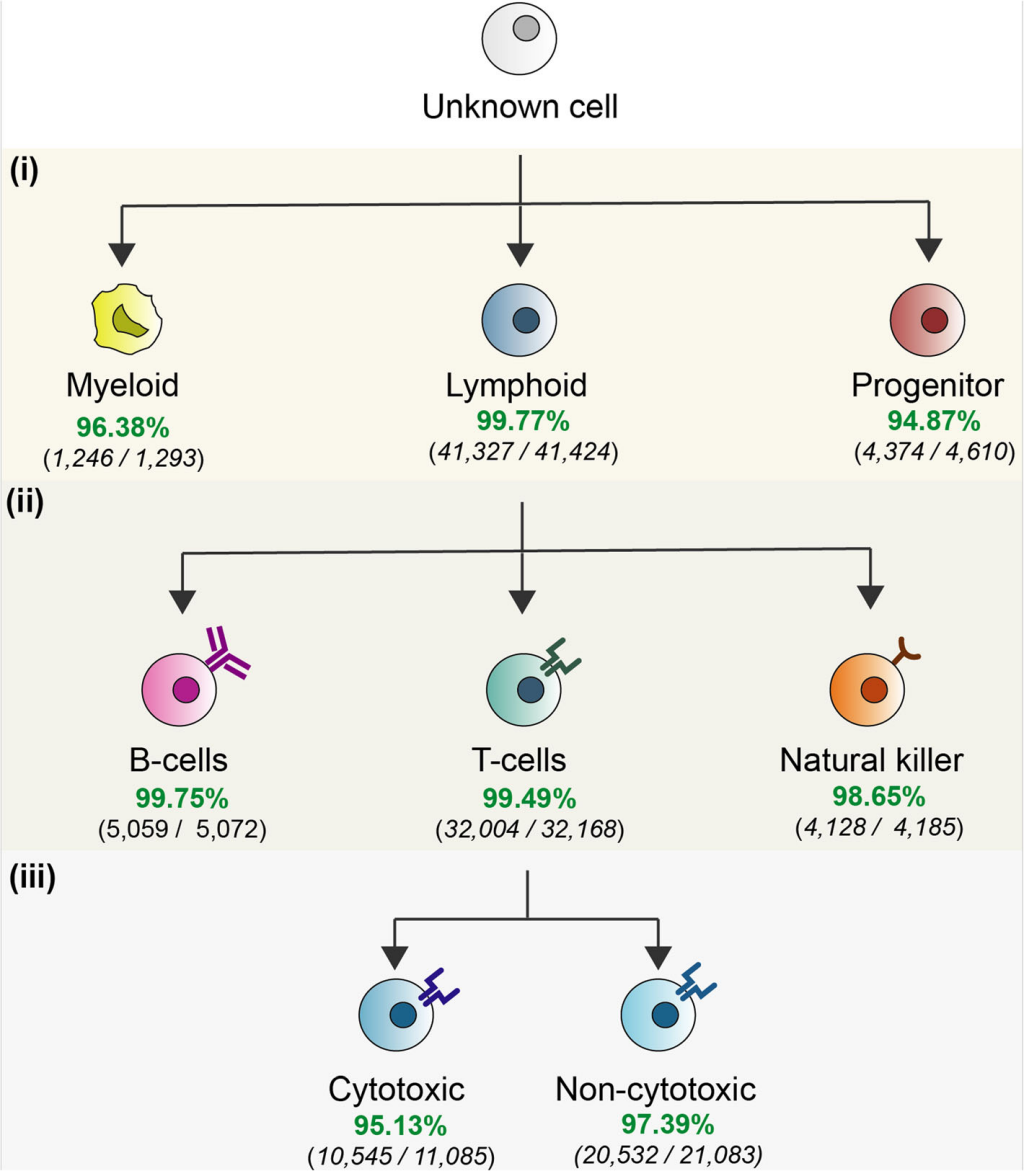
Prediction results of PBMCs. The average number of cells for each cell type across all ten bootstrap replicates is shown. (i) First, every single cell was classified as myeloid, lymphoid, or blood progenitor. (ii) A second layer of prediction is used to classify all lymphoid cells as B cells, T cells, or natural killer. (iii) Finally, all T cells are subclassified as cytotoxic or non-cytotoxic. Confidence intervals for mean estimates are included in Additional file 2: Table S12

Peripheral blood mononuclear cells (PBMCs) comprise diverse groups of cells that are extensively studied because of their role in mediating adaptive and innate immune responses as well as their implication in autoimmune, metabolic, and infectious diseases [37, 38]. Here, we aimed to classify PBMCs from which their cell identity was determined based on fluorescence-activated cell sorting (FACS) [39]. For doing so, we developed a hierarchical tree-based prediction approach in which individual cells are classified along with categories following the hematopoietic lineage of PBMCs (see Fig. 4). This strategy decomposes the problem of predicting closely related cells by training models for cell types that are comparable in terms of their variance and hierarchic organization in the hematopoietic lineage. Firstly, a cell is classified as myeloid, lymphoid, or progenitor. Secondly, all cells predicted as lymphoid are further subcategorized into B cells, T cells, and Natural Killer. Finally, the cytotoxic state of the predicted T cells is assigned. For each level in the hierarchy, a *scPred* model was trained. To verify the performance of this approach, we compared the cell type information derived from FACS versus the predictions made by *scPred* based exclusively on the transcriptome. We performed ten bootstrap replicates to estimate to test the performance of our approach by splitting the 94,655 PBMCs into training and test groups. Overall, 97.67% of the cells from the test group (45, 884 out of 47, 328) were classified correctly (see Fig. 4). Notably, the highest and lowest accuracies obtained for a cell-type group were 99.7% and 95.13% for lymphoid cells and cytotoxic T cells respectively. Bootstrap 95% confidence intervals are reported in Additional file 1: Table S12. These results demonstrate that *scPred* can accurately classify cells that share very similar transcriptional profiles.

### Accurate prediction of human dendritic cells from data generated across laboratories

We next sought to evaluate the performance of *scPred* when the training and testing cells sequenced using the same protocol but in different laboratories. For developing single cell-based diagnostic tests, this is an important consideration, as in the majority of cases a predictive model will be developed using sequence data generated from different laboratories to those conducting testing. Between-site effects could bias the predictive performance of a test if the between-site batch effects are confounded with the model classification features. While between-site variance for bulk-RNA-sequencing is small [40], it has not yet been fully evaluated for scRNA-Seq. We chose to evaluate the performance of *scPred* by building a prediction model to identify dendritic cells from peripheral blood samples [1]. Dendritic cells are antigen-presenting cells, and their main function is to process antigen material and present it on the cell surface to T cells, acting as messengers between the innate and adaptive immune systems. Using the cell type classification based on scRNA-seq and flow validation given in Villani et al. [1], we built a *scPred* prediction model using scRNA-seq data generated using the SMART-seq2 protocol for 660 dendritic cells. The best fit model from *scPred* used 11 PCs, which collectively explained 5.97% of the variance in the entire training data cohort.

**Fig. 5:**
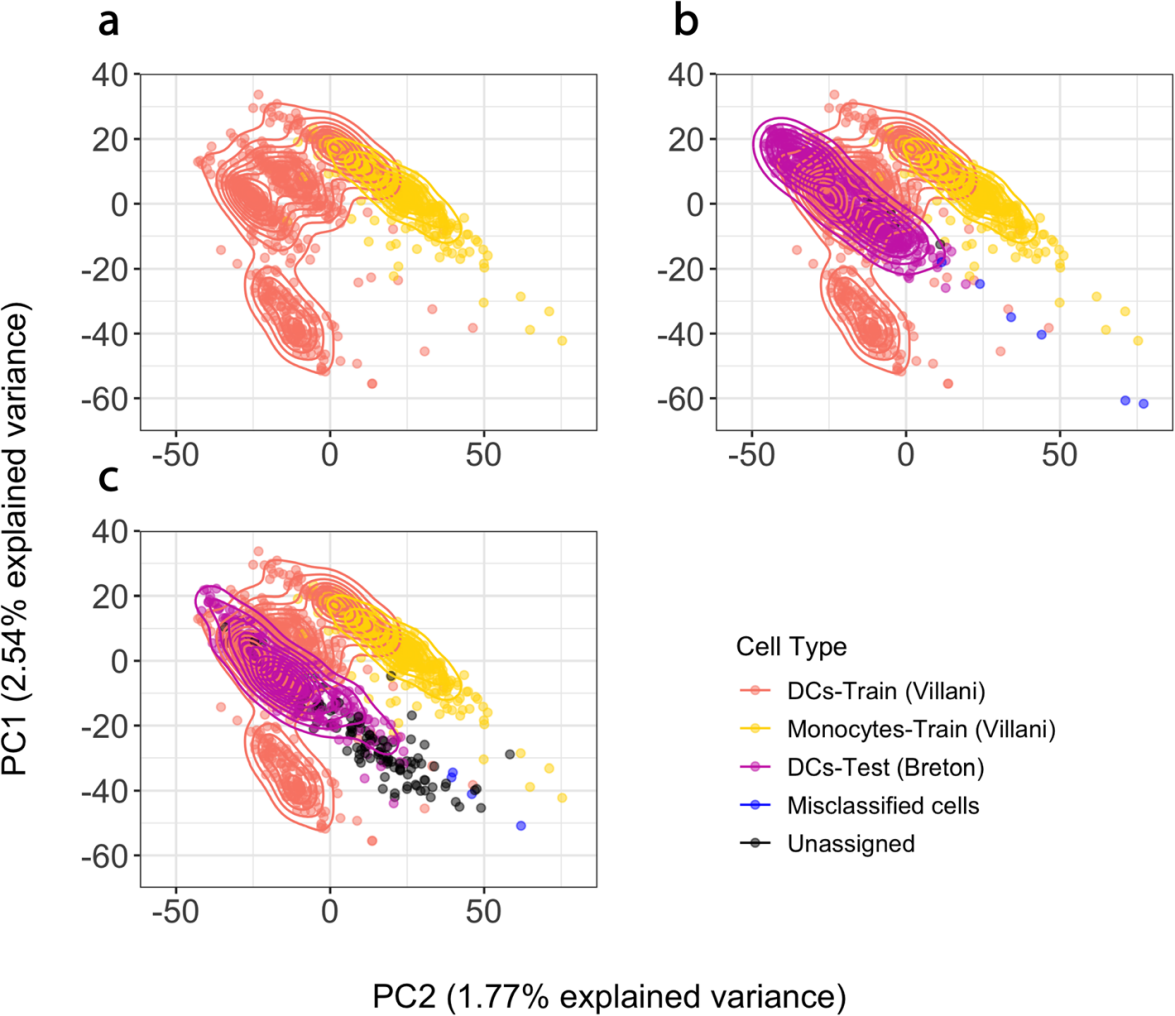
Prediction of human dendritic cells. **a** The training dataset (Villani et al.) of dendritic cells and monocytes was eigendecomposed (orange and yellow points and density lines). **b** Dendritic cells from the test dataset (Breton et al) were projected onto the training eigenspace (purple points). scPred predicted 98% of dendritic cells derived from peripheral blood correctly and 82% from umbilical cord (Breton et al.). Blue points correspond to cells that were misclassified and black points to unassigned cells

We then applied our model to predict dendritic cells from two independent test data cohorts consisting of scRNA-seq data from a heterogeneous mix of cells from peripheral blood (461 cells) and umbilical cord (420 cells), also generated using the SMART-Seq2 protocol in a different laboratory [41]. Notably, the accuracy for peripheral blood-derived cells was 98% (Table 2, Fig. 5 and Additional file 2: Table S13). When we applied the *scPred* model to the cells obtained from an umbilical cord, the overall accuracy was 82%. This lower prediction accuracy possibly reflects a contamination or incorrect original classification of cells obtained from the umbilical cord. To evaluate this, we looked for differentially expressed genes between the 60 cells with a dendritic cell class probability of < 0.9 and the remaining cord cells (see Additional file 3: Table S14). We identified upregulation of genes overlapping the T cell receptor gamma locus: TRGC2, TARP, and X06776 (a truncated mRNA from the TRG gamma gene). Additionally, an over-representation of myeloid and neutrophil-related biological processes for upregulated genes was identified in these cells (see Additional file 3: Table S15). All gene ontologies corresponded to myeloid cells, and the presence transcripts from a T cell specialized locus suggests the presence of T cells or alternatively greater heterogeneity in cord-derived cells. Collectively, these results demonstrate that *scPred* is able to accurately predict cell classes using a model trained on data generated in a different laboratory to the test data, without the need to normalize data between sites. This implies that any potential batch effects, or laboratory effects, are not captured in the informative features used to develop the prediction model.

**Table 2:**
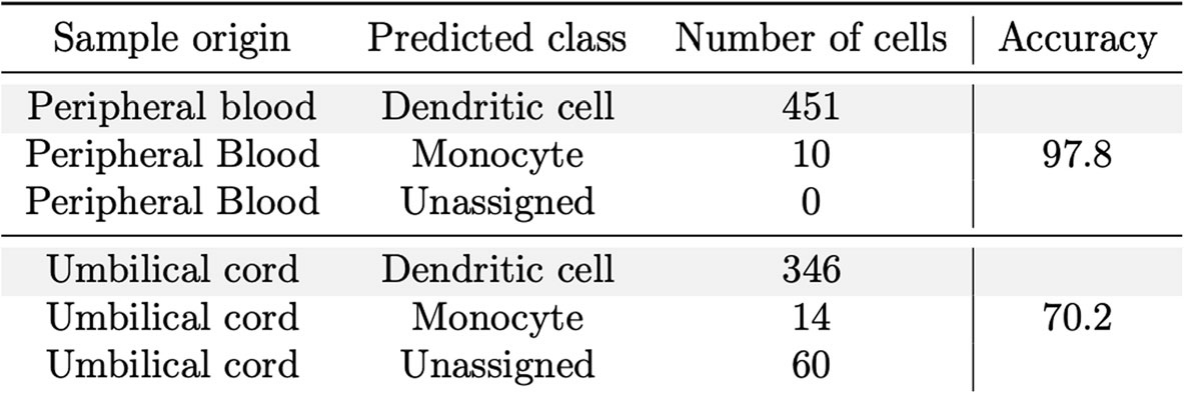
Prediction of dendritic cells from Breton et al. test data. The first column corresponds to the sample origin of the dendritic cells analyzed by Breton et al. The second column shows the class label assigned by scPred. The accuracy is reported by sample origin. Importantly, only 10 dendritic cells out of 461 were classified as monocytes. This demonstrates the high accuracy achieved by scPred to distinguish dendritic cells from monocytes from peripheral blood. For umbilical cord derived-cells, only 14 out of 420 cells were classified as monocytes and 60 were unassigned as their probability to belong to any of the classes from the training set was low. As discussed in the main text, we argue that these cells correspond to other cell subtypes

### Accurate classification when cell types are imbalanced

**Fig. 6:**
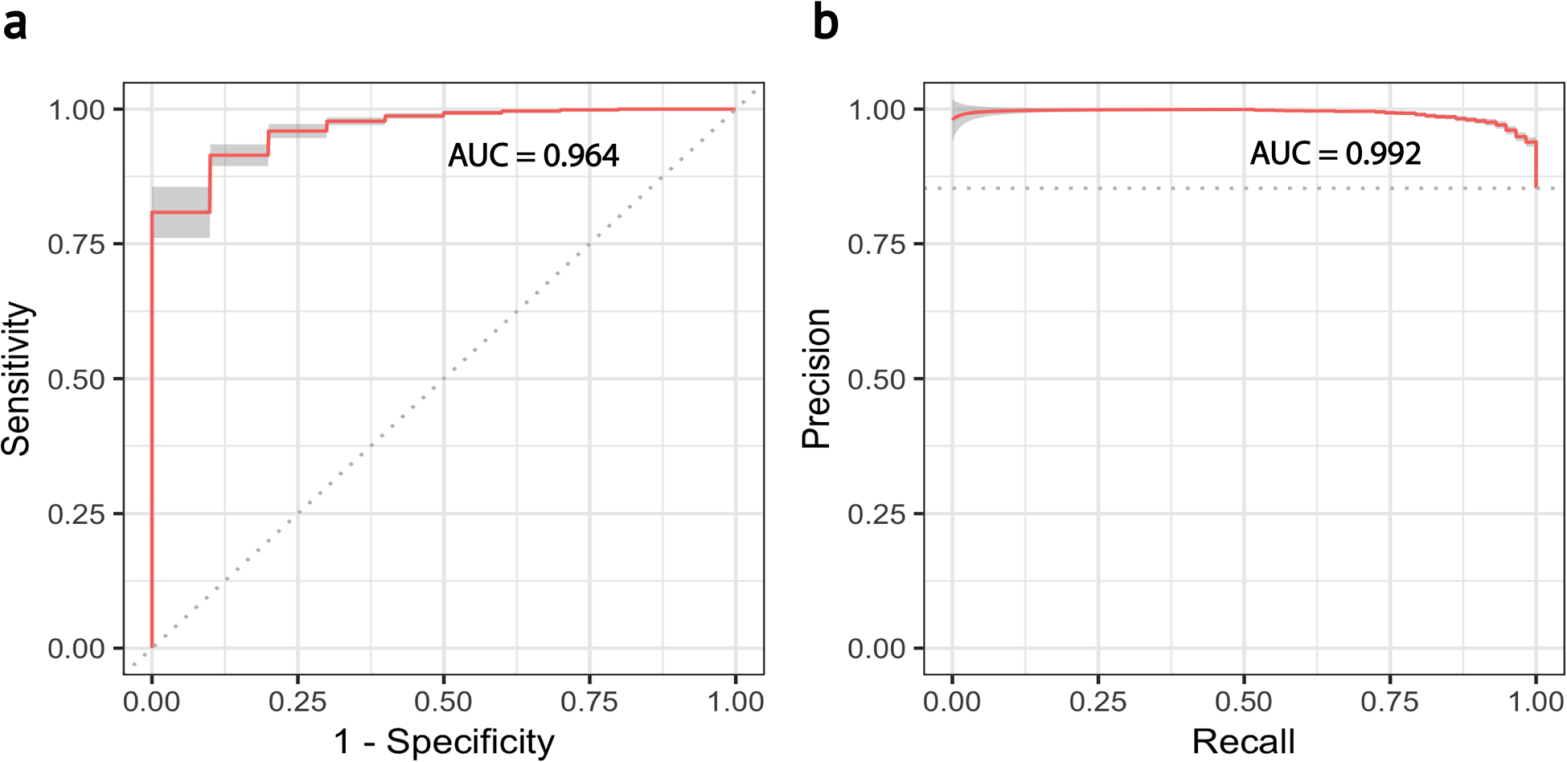
Prediction results of colorectal cancer epithelial stem/TA-like cells. The performance of the prediction was measured using the receiver operating characteristic area under the curve (ROC AUC) and the precision-recall area under the curve (PR AUC). 95% confidence bands are shown in both cases for 50 bootstrap replicates. **a** ROC AUC. The area under the curve shows the relationship between the cells incorrectly assigned to that come from tumor samples versus the ones that were correctly assigned by the prediction model as tumor cells using a series of different threshold points. **b** PR AUC. The area under the curve measures the relationship between the cells correctly classified as tumor cells versus the fraction of cells correctly assigned as tumor cells from the total number of cells classified as tumor cells. An AUC value of 0.992 shows robustness to class imbalance

Primary tumors contain cells that are both tumor and non-tumor cells of varying types. However, importantly, tumor cells originate from the same cell subtypes of one or more of the original healthy cells in a tissue. Numerous methods exist for classifying (or diagnosing) a whole tissue biopsy as either cancerous or non-cancerous based on DNA genotyping [42], transcriptome profiling [43, 44], or histochemistry [45, 46]. Most of these methods work well, but are unable to accurately classify heterogeneity at a cellular level, and do not work if the percentage of tumor cells in a biopsy is small. We applied *scPred* to predict epithelial tumor cells from a heterogeneous population of cells from tumor- and normal mucosa-matched samples from 11 colorectal cancer patients [47]. Of the 275 cells from colorectal cancer samples, the imbalance in the proportions of colorectal cancer epithelial stem/TA-like cells compared to healthy controls was a 1:5 ratio of normal to tumor cells. The prediction accuracy was evaluated using a bootstrapping method, training on a randomly sampled 75% of the data and predicting on the remaining 25%, while correcting for class imbalance using the smote algorithm [48]. To estimate the variance of prediction accuracy, 50 bootstraps were performed, and the mean across replicates was calculated (see the “Methods” section). Overall, the mean area under the receiver-characteristic function was 96.4 with 95% confidence intervals of 95.5–97.2 (Fig. 6a). Likewise, the mean precision-recall curve was 0.992 (95% confidence intervals of 0.989–0.995) (Fig. 6b). Given the imbalance in the proportions of colorectal cancer, the high area under the precision-recall curve and small confidence intervals indicate that *scPred* is robust to class imbalance in the training data. The high specificity of the model under this scenario implies that a single-cell prediction method would be able to accurately diagnose disease status using scRNA-seq data from a limited number of cells. For example, here, the mean sensitivity for tumor cells is 0.761 and the specificity is 0.958. Thus, if in a patient sample 100 cells were single-cell sequenced, the probability of incorrectly classifying 10 cells as a tumor cell from a healthy individual would be 6.3 × 10^−7^. Conversely, once 10 cells are correctly classified tumor cells in a true tumor biopsy, the probability of accurately diagnosing the disease state is approximately 1.

## Discussion

Single-cell RNA sequencing has provided the ability to analyze the transcriptomic profile of individual cells, leading to the identification of novel cell types and the characterization of heterogeneous cell populations. Here, we introduced *scPred*, a novel method to classify single cells based on singular value decomposition and a support vector machine model. *scPred* takes advantage of the informative signals spread across orthonormal linear combinations of the gene expression values and minimizes the incorporation of noise to the prediction model by excluding principal components with a low contribution to the variance explained. *scPred* uses support vector machines as a default machine learning approach as it is suitable for large datasets and accounts for various sources of data [49]. Supervised machine learning methods have been used in previous studies to classify various cell types such as retinal bipolar cells [50] and embryonic stem cells [51]. Here, we have shown that *scPred* shows high accuracy in a variety of relevant biological and clinical scenarios which include predicting pathological cell states from gastric and colorectal cancer as well as characterizing the cell-type composition of peripheral blood mononuclear cells. Collectively, our results show that *scPred* is able to accurately classify individual cells from an independent sample to those used to train the prediction model. However, the ability to do so even when using a training cohort of cells whose scRNA-seq data is assayed from different platforms has important implications for a practical implementation of *scPred*. The ability to build a single-cell training cohort using data generated from multiple platforms means that composite reference datasets can be generated, which will increase the predictive accuracy of *scPred* through a more accurate estimate of the model effects. One of the advantages of *scPred* is that by reducing the dimensions of the gene expression matrix via singular value decomposition we also decrease the number of features to be fit, reducing both the computational requirements for prediction and the prediction model parameter space. While we have used a support vector machine method, the *scPred* software can be easily adjusted to use other classification algorithms [52], allowing a user to choose the models that suit the effect distributions of their data best.

## Methods

The *scPred* method is split into two major steps. First, a prediction model is built using a training dataset of scRNA-seq data. The second step is the application of this prediction model to scRNA-seq data obtained from independent sample, with each cell then assigned a probability of belonging to a given class based on the fit of its scRNA-seq expression levels in the prediction model. Below, we have outlined the methods for each of these steps. We start with a single-cell gene expression matrix *C*_Train_ (CPM values—count per million mapped reads) obtained from different characterization classes: for example, from different cell subtypes, cells obtained from disease versus control samples, or cells defined as different states.

### Training step

The training expression matrix is log2-transformed log_2_(*G*_Train_+1) to linearize the expression values for each gene and stabilize the variance across a large expression range. Let *G*_Train_ be the log_2_-transformed expression matrix *C*_Train_ with *n* single cells and *m* genes:

$$ {G}_{\mathrm{Train}}=\left[\begin{array}{ccccc}{x}_{11}& {x}_{12}& {x}_{13}& \cdots & {x}_{1m}\\ {}{x}_{21}& {x}_{22}& {x}_{23}& \cdots & {x}_{2m}\\ {}\vdots & \vdots & \vdots & \ddots & \vdots \\ {}{x}_{n1}& {x}_{n2}& {x}_{n3}& \cdots & {x}_{nm}\end{array}\right] $$

We subsequently center and scale *G*_Train_ using the mean and standard deviation of gene expression values of each gene, calculated using the following formulas:

$$ \mu =\frac{1}{n}\sum \limits_{i=1}^n{x}_1\kern1.00em \sigma =\sqrt{\frac{1}{n-1}\sum \limits_{i=1}^n{\left({x}_i-\mu \right)}^2} $$

Each mean is subtracted from all *m*_*th*_ elements of their corresponding *n*_*th*_ row, and the result is divided by the respective standard deviation as follows:

$$ M=\left[\begin{array}{ccccc}\left({x}_{11}-{\mu}_1\right)/{\sigma}_1& \left({x}_{12}-{\mu}_2\right)/{\sigma}_2& \left({x}_{13}-{\mu}_3\right)/{\sigma}_3& \cdots & \left({x}_{1m}-{\mu}_m\right)/{\sigma}_m\\ {}\left({x}_{21}-{\mu}_1\right)/{\sigma}_1& \left({x}_{22}-{\mu}_2\right)/{\sigma}_2& \left({x}_{23}-{\mu}_3\right)/{\sigma}_3& \cdots & \left({x}_{2m}-{\mu}_m\right)/{\sigma}_m\\ {}\vdots & \vdots & \vdots & \ddots & \vdots \\ {}\left({x}_{n1}-{\mu}_1\right)/{\sigma}_1& \left({x}_{n2}-{\mu}_2\right)/{\sigma}_2& \left({x}_{n3}-{\mu}_3\right)/{\sigma}_3& \vdots & \left({x}_{nm}-{\mu}_m\right)/{\sigma}_m\end{array}\right] $$

We next calculate orthogonal vectors for the gene expression values using a singular value decomposition (SVD) method. To do so, the matrix *M* needs to be factorized into the product of three matrices as follows:

$$ M=U\sum {V}^T $$

where *U* and *V* are orthonormal matrices and Σ a diagonal matrix.

First, we compute the product *MM*^*T*^. To find *U*, we orthogonally diagonalize *MM*^*T*^.

$$ M{M}^T=\left(U\sum {V}^T\right)\left(V{\sum}^T{U}^T\right)=U\sum {\sum}^T{U}^T= UD{U}^{-1} $$

Then, *U* contains the eigenvectors of *MM*^*T*^ (or left singular vectors of *M*) and *D* its eigenvalues.

$$ U=\left[\begin{array}{ccc}{c}_{11}& \cdots & {c}_{1n}\\ {}\vdots & \ddots & \cdots \\ {}{c}_{1n}& \cdots & {c}_{nn}\end{array}\right] $$

Similarly, to calculate *V*, we compute the product *M*^*T*^*M* and diagonalize *M*^*T*^*M* to calculate its eigenvectors and eigenvalues.

$$ {M}^TM=\left(V{\sum}^T{U}^T\right)\left(U\sum {V}^T\right)=V{\sum}^T\sum {V}^T= VD{V}^{-}1 $$

*V* contains the eigenvectors of *M*^*T*^*M* (or right singular vectors of *M*) and *D* its eigenvalues.

$$ V=\left[\begin{array}{ccc}{v}_{11}& \cdots & {v}_{1m}\\ {}\vdots & \ddots & \vdots \\ {}{v}_{1m}& \cdots & {v}_{mm}\end{array}\right] $$

Σ is a diagonal matrix with the squared root eigenvalues of *M*^*T*^*M* (or singular values of *M*) along the diagonal.

$$ \sum =\left[\begin{array}{ccc}{s}_{11}& & \\ {}& \ddots & \\ {}& & {s}_{nm}\end{array}\right] $$

The matrix product *U*Σ gives the principal components (PCs) or “scores”, which are a new set of uncorrelated linear variables that capture the maximum variance from the single-cell expression matrix *M*. The individual squared values of the diagonal entries of Σ divided by the sum of all squared values give the variance explained by each principal component. PCs are in descending order according to the variance each of them explains.

$$ S=U\sum $$

We next identify the PCs whose scores have significant differences between the classification cell classes. We initially create a subspace of *S* (namely *R* with *n* rows and *r* columns -dimensions-), such that each dimension explains at least 0.01% of the variance of the matrix *M*. However, it is important to note that at this stage, we do not select features to fit in a prediction model. To identify the informative dimensions, a two-tailed Wilcoxon rank-sum test is performed for each PC to assess whether there is a significant difference in the distributions of PC scores for cells in different classes. The resulting *p* values are adjusted for multiple testing using a Benjamini-Hochberg false discovery rate correction. Columns from *R* are ranked in ascending order based on their corresponding *p* values. This step allows us to identify PCs with the largest difference in their distributions of the scores between the classes, and thus is expected to be the most informative as features used as input predictors in a classification model.

From *R*, we create a subspace *F* with only *f* columns with associated adjusted *p* values less than 0.05. The columns of *F* are used as features to train a support vector machine model with a radial kernel. A support vector classifier consists of a subspace (called hyperplane) of dimension *h*-1 with regard to its ambient high dimensional space *H* with *h* dimensions, which linearly separates the observations (cells) according to the class they belong to. A margin around the hyperplane is defined in order to minimize the misclassifications. The width of the margin is determined by observations called *support vectors*. Here, we find a hyperplane that separates single cells based on their PC scores into the classification classes. Those cells that define the margin can be thought of as *supporting cells* of the hyperplane.

When the observations cannot be separated in the feature space using a linear boundary, a “kernel trick” is used to map observations into a high-dimensional space where they can be linearly separated by a hyperplane. Let Φ be a function that maps single cells from a *F* space of *f* dimensions to a higher dimensional space *H*.

$$ \varPhi :F\to H $$

And *k*(*x*, *x*_1_) be a kernel function that returns the inner product of the images of two cells (based on the values of the *f* principal components in *F*).

*k*(*x*, *x*_1_) = 〈*Φ*(*x*), *Φ*(*x*_*i*_)〉

However, instead of computing a feature map Φ for all observations, the following shortcut is possible using a Gaussian radial basis kernel [53]:

$$ \left\langle \varPhi (x),\varPhi \left({x}_i\right)\right\rangle =\mathit{\exp}\left(-\sigma \parallel x-{x}_1{\parallel}^2\right) $$

where *σ* is a constant greater than zero estimated via cross-validation. Thus, Eq. (7) can be rewritten as:

$$ k\left(x,{x}_i\right)=\mathit{\exp}\left(-\sigma \parallel x-{x}_i{\parallel}^2\right) $$

Hence, the coordinates of the cells in *H* are not computed.

Then, we can define a function *f*(*x*) that returns a decision value which indicates whether a cell belongs to a class or the other using the kernel function.

$$ f(x)=\sum \limits_{i=1}^n{\alpha}_ik\left(x,{x}_i\right) $$

*α*_i_ parameters are estimated by solving the following minimization problem:

$$ t\left(w,\xi \right)=\frac{1}{2}\parallel w{\parallel}^2+\frac{C}{n}\sum \limits_{i=1}^n{\xi}_i $$

subject to

$$ {\displaystyle \begin{array}{ccc}{\forall}_i\in \left\{1,\dots, n\right\}& & {y}_i\left(\left\langle {x}_i,w\right\rangle +b\right)\ge 1-{\xi}_i\\ {}& {\xi}_i\ge 0& \end{array}} $$

And being the hyperplane defined by the following set:

$$ \left\{x\in H|\left\langle w,x\right\rangle +b=0\right\} $$

*w* is a weight vector in the feature space *N* perpendicular to the hyperplane which helps to define the margin, *ξ* is a slack variable that allows each cell to be on the wrong side of the hyperplane or the margin in order to deal with outliers, *n* is the number of observations (cells), *y*_*i*_ is a variable that indicates whether the cell *x*_*i*_ belongs to one class (*y* = 1) or the other (*y* = − 1), and *C* is a cost parameter that penalizes the sum of *ξ*_*i*_ . As *C* increases, the margin becomes wider and more tolerant of violations by cells. By enlarging the feature space using a polynomial kernel, the cells are linearly separated in *H* [54]. To train the model, we determine the cost *C* and *σ* parameters via cross-validation and select the values that maximize the prediction performance. Finally, class probabilities are calculated using a sigmoid function fitted on the decision values returned by the classifier *f*(*x*) [53].

$$ \Pr \left(y=1|f\right)=\frac{1}{1+{e}^{Af+B}} $$

The final trained model consists of a set of parameters that maximizes the margin between the training observations and the hyperplane in order to separate single cells according to their classification class. K-fold cross-validation is performed as described in the *caret* package [52]. If the number of classes is more than two for the response variable, then *n* binary classification models are trained. For each classification model, we categorized all cells into two classes depending on the class being studied: *positive class* (cell type(s) of interest) and *negative class* (remaining cell types), “one-versus-all” approach.

### Prediction step

Once the model has been trained and evaluated, it can be used to classify single cells from an independent dataset from which the cell classes are unknown. Here, we apply the trained model(s) to classify cells from a testing dataset.

Given a test expression matrix *C*_Test_ with *n* single cells as rows and *m* genes as columns, let *G*_Test_ be the log_2_-transformed expression matrix *C*_Test_:

$$ {G}_{\mathrm{Test}}=\left[\begin{array}{ccccc}{x}_{11}& {x}_{12}& {x}_{13}& \cdots & {x}_{1m}\\ {}{x}_{21}& {x}_{22}& {x}_{23}& \cdots & {x}_{2m}\\ {}\vdots & \vdots & \vdots & \ddots & \vdots \\ {}{x}_{n1}& {x}_{n2}& {x}_{n3}& \cdots & {x}_{nm}\end{array}\right] $$

The matrix is centered and scaled using the means and variances calculated from *G*_Train_:

$$ {M}_{\mathrm{Test}}=\left[\begin{array}{ccccc}\left({x}_{11}-{\mu}_1\right)/{\sigma}_1& \left({x}_{12}-{\mu}_2\right)/{\sigma}_2& \left({x}_{13}-{\mu}_3\right)/{\sigma}_3& \cdots & \left({x}_{1m}-{\mu}_m\right)/{\sigma}_m\\ {}\left({x}_{21}-{\mu}_1\right)/{\sigma}_1& \left({x}_{22}-{\mu}_2\right)/{\sigma}_2& \left({x}_{23}-{\mu}_3\right)/{\sigma}_3& \cdots & \left({x}_{2m}-{\mu}_m\right)/{\sigma}_m\\ {}\vdots & \vdots & \vdots & \ddots & \vdots \\ {}\left({x}_{n1}-{\mu}_1\right)/{\sigma}_1& \left({x}_{n2}-{\mu}_2\right)/{\sigma}_2& \left({x}_{n3}-{\mu}_3\right)/{\sigma}_3& \cdots & \left({x}_{nm}-{\mu}_m\right)/{\sigma}_m\end{array}\right] $$

and *M*_Test_ is projected onto the training PCA coordinate basis using the rotation matrix *V* after log2-transforming and scaling the data according to the training feature space:

$$ P={M}_{\mathrm{test}}V $$

*P* contains the projection of the single cells from test dataset onto the PCs from the training data. Informative PCs listed in the *R* training subspace are extracted from *R* and used as features to predict the classification classes of the cells from the test dataset using the trained support vector machine model (see Fig. 1).

If more than two models were trained, all cells in *P* are classified using the *c* trained models. If the maximum probability obtained across all models is greater than a threshold (0.9 by default), the cell is labeled according to the positive class corresponding to model the highest probability; otherwise, the cell is labeled as “Unassigned.”

### Predicting cell type from scRNA-seq data using *scPred*

Our *scPred* method provides a generalized framework to classify a given cell based on its gene expression values. Importantly, our method is designed to solve the problem of individual gene feature selection and enable subtle effects spread across many genes to be utilized through orthogonal components of variance. In doing so, we anticipate an increase in the prediction performance over current gene-centric feature selection, as *scPred* will incorporate the small effects of many genes. To demonstrate both the utility and performance of *scPred*, we first validated the performance against an orthogonal molecular assay and then addressed three distinct biological examples of classification of single cells: firstly, by predicting specific α, β, δ, and γ cell subtypes from pancreas islets of Langerhans; secondly, classifying dendritic cells using a heterogeneous mix of single cells as a reference; and finally, identifying the presence of cancer cells from a heterogeneous composition of cells from whole tissue in both tumors and matched healthy controls. For all datasets, we removed all cells above or below 3 median absolute deviations (MAD) from the median library size, mitochondrial, and ribosomal gene expression. Furthermore, all genes with zero counts across all cells and genes not expressed in at least 1% of the whole population were discarded. Finally, all count matrices were transformed to CPM values, and genes being expressed more than five CPM were preserved. *scPred* predicts cells using a default probability threshold of 0.9 to ensure high confidence in the classification. All cells below this threshold for each cell type are labeled as unassigned. Here, we define sensitivity as the proportion of cells that were correctly classified for the cell type of interest, based on the default threshold with respect to the remaining cells that do not belong to this cell type (i.e., number of cells correctly classified from the positive class/(cells correctly classified from the negative class + unassigned cells that belong to the positive class). Likewise, specificity is the proportion of the cells from the negative class that were correctly classified with respect to the remaining cells from that class and the unassigned cells that belong to the negative class). Therefore, for the case of binary classification, sensitivity and specificity can both be zero if all cells from the positive and negative classes had a probability lower than 0.9 for each class respectively. The area under the AUROC and AUPRC were determined using the MLmetrics R package from CRAN https://cran.r-project.org/web/packages/MLmetrics/index.html.

### Gastric cancer tumor versus non-tumor prediction

The collection of this data was conducted in compliance with the Helsinki Declaration. The institutional review board at Stanford University School of Medicine approved the study protocol (19071), and informed consent was obtained. We collected two matched sets of two samples including gastric primary cancer, and normal stomach tissue. Tissue biopsies were obtained from surgical resection of a primary gastric adenocarcinoma and matched adjacent normal tissue. Immediately 10 min after resection, the tumor sample was stored in RPMI medium on ice for less than 1 h. The samples were then microdissected and dissociated into a cellular suspension by the gentleMACS Octo Dissociator as per the manufacturer’s recommendations and the 37C_h_TDK_3 program (Miltenyi Biotec, Bergisch Gladbach, Germany). Single-cell RNA-seq was performed after thawing cryopreserved sample stored in liquid nitrogen in DMSO. Histopathology of this gastric cancer revealed moderate to poorly differentiated features with a 60–70% tumor fraction. Immunohistochemistry demonstrated a loss of MLH1 and PMS2 expression. The loss of these proteins indicated that this tumor had microsatellite instability (MSI) where cancer cells have a hypermutable state because of loss of DNA mismatch repair. The tumor tissue was disaggregated into a single-cell suspension and analyzed scRNA-seq. We used the Chromium Controller instrument (10X Genomics Inc., Pleasanton, CA) and the Single Cell 3′ Reagent kit (v2) to prepare individually barcoded scRNA-seq libraries following the manufacturer’s standard protocol. Briefly, single-cell suspensions were loaded on a Chromium and were partitioned in droplets. Reverse transcription is performed, followed by droplet breaking, and cDNA amplification. Each cDNA molecule thus contained the read 1 sequencing primer, a 16-bp cell-identifying barcode, and a 10-bp UMI sequence. We performed enzymatic fragmentation, end-repair, and A-tailing followed by ligation of a single-end adapter containing the read 2 priming site. Finally, sequencing libraries were quantified by qPCR before sequencing using 26 × 98 paired-end reads. The Cellranger software suite was used to process scRNA-seq data, sample demultiplexing, barcode processing, and single-cell 3′ gene counting. Cellranger provided a gene-by-cell matrix, which contains the read count distribution of each gene for each cell. The gene expression matrix was split according to the disease status of each cell (defined by the presence of microsatellite instability) to create a training dataset. We selected only class informative PCs explaining at least 0.01% of the variance and using an adjusted alpha threshold of 0.05. Tenfold cross-validations were performed to train a support vector machine model with a radial kernel. The trained model was applied to independent cells to evaluate classification accuracy. To train two of the baseline models using SVM, we set all the coefficients of the hyperplane to 0 and 1 respectively. We trained a model using all principal components to evaluate the performance of the feature selection of *scPred*. Differentially gene expression analysis was performed using edgeR [55] to obtain discriminant genes between tumor and non-tumor cells to be used as predictors. Ten bootstrap replicates were performed for all baseline models using the same dataset partitions from the main prediction analysis using *scPred*.

### Prediction of islets of Langerhans subtypes

We considered three independent datasets to train a prediction model to classify α (alpha), β (beta), δ (delta), and γ (gamma) cell subtypes: Muraro et al. [25] consisting of 1522 cells from a CEL-Seq2 protocol, Segerstolpe et al. [3] consisting of scRNA-seq data from 1321 cells generated using the Smart-Seq2 protocol, and 1349 cells from Xin et al. [26] whose gene expression levels were assayed using the SMARTer protocol (see Additional file 2: Table S1). We integrated the three datasets using the intersection of genes between them and obtained a single aggregated matrix. We applied the Seurat alignment approach [30] to account for technical differences across the different datasets used for the training dataset. First, we determined the most variable genes (528) in at least two of the three datasets to compute the loadings and the first 30 PCs using the implicitly restarted Lanczos bidiagonalization algorithm [56]. Then, we used the loadings from the training eigendecomposition to project the testing dataset (Baron et al.) and obtained the cell embeddings. After the alignment, no batch effect was observed (see Fig. 3). Then, we trained a prediction model considering only class-informative PCs using a multiple testing corrected alpha level of 0.05 using (see *scPred* in the “Methods” section) using the scores from the aligned training eigenspace only. Tenfold cross-validations were performed to train a support vector machine model with a radial kernel. To assess the performance of our prediction model, we predicted the specific cell types of 7932 cells using their scRNA-seq data generated using the inDrop protocol [31]. In addition to our main analysis, we trained a prediction model using the Baron dataset only as a training reference and applied the model to classify cells from the Muraro, Segerstolpe, and Xin datasets respectively using the same methodology described above. We then compared the prediction performance of *scmap* [33], *caSTLe* [34], *singleCellNet* [35], and *scID* [36] following the same approach. To train the classifiers using *scmap*, we used 500 genes as features. Cluster and cell projections were performed using both algorithms. For the *caSTLe* cell classification, we reproduced the pipeline code from https://github.com/yuvallb/CaSTLe using the Baron data as source dataset and the Muraro, Segerstolpe, and Xin as the target dataset. Code for *singleCellNe*t and *scID* was obtained from https://github.com/pcahan1/singleCellNet and https://github.com/BatadaLab/scID respectively.

### Prediction of peripheral blood mononuclear cells

We used the dataset obtained by Zheng et al. [39] to train and test prediction models to deconvolute the cell-type identity of peripheral blood mononuclear cells. We defined three layers of prediction based on the hierarchy of PBMCs in the hematopoietic lineage and trained a *scPred* model for each of them using a training partition from the dataset. Then, every single cell from the test fold was classified as follows: the first model classifies the cell as myeloid, lymphoid, or blood progenitor based on a feature eigenspace including all these cells. Furthermore, all cells classified as lymphoid are subcategorized into B cell, T cell, or Natural Killer using a second classifier trained to distinguish between these classes. Finally, predicted T cells are subclassified into cytotoxic and non-cytotoxic. Ten bootstrap replicates were performed to calculate a 95% confidence interval of the accuracies for every cell type.

### Prediction of dendritic cells: training data of dendritic cells and monocytes

scRNA-seq data was obtained using a Smart-Seq2 protocol [1]. After quality control, 660 dendritic cells and 335 monocytes were used to train a prediction model applying a 0.01% variance-explained filter and a corrected alpha level of 0.05 to select the informative PCs. Tenfold cross-validations were performed to train a support vector machine model with a radial kernel. We tested the *scPred* prediction model against dendritic cells from an independent study [41], whose scRNA-seq data had been generated using the SMART-Seq 2 protocol. After quality control, 150 primary human conventional dendritic cells (cDCs), 420 cord blood pre-cDCs, and 311 blood pre-cDCs were kept. The final training model consisted of only 11 discriminant PCs explaining 5.97% of the variance from the Villani et al. dataset. The training error of the model was 0.018, and 232 cells were used as support vectors. Differential expression analysis between the unassigned cells and remaining cells from cord blood was performed using edgeR [55]. Genes with a log fold-change greater or lower than 2 and an adjusted *p* value less than 0.05 were considered as differentially expressed. Gene ontology analysis was performed using http://pantherdb.org/.

### Prediction of colorectal cancer cells

We obtained the human colorectal cancer dataset under the GEO accession number GSE81861. We analyzed only the stemTA cell subtype as they are the most abundant epithelial subpopulation. After quality control, 275 cells stemTA cells and 21,933 genes were kept. The gene expression matrix was split according to the sample origin of each cell (tumor or normal) to create a training and testing dataset such that the former partition contained 75% of cells and the latter 25%. To create the partitions, we used the SMOTE algorithm [48] to account for class imbalance. We selected only class-informative PCs explaining at least 0.01% of the variance and using an adjusted alpha threshold of 0.05. Tenfold cross-validations were performed to train a support vector machine model with a radial kernel. Finally, we obtained the areas under the ROC and precision-recall curves to measure the performance of *scPred*. Ten bootstrap replicates were performed.

## Supplementary information

**Additional file 1: Figure S1.** Expression of EPCAM, MLH1, and TFF3. **Figure S2.** Effect of the sequencing depth on prediction performance of gastric tumour cells. **Figure S3.** Effect of the number of cells on prediction performance of gastric tumour cells. **Figure S4.** Distribution of conditional class probabilities for single cells from the Baron test dataset across all four models.

**Additional file 2: Table S1.** Classification performance comparison between scPred and other prediction baseline methods. For all methods, results are reported using the same data set partitions across all bootstrap replicates. **Table S2.** Summary of pancreas datasets. Training dataset consisted of 4292 cells and 32 human samples in total. **Table S3.** Prediction results of pancreatic cells from Baron dataset. **Table S5.** Prediction performance of pancreatic cells from Baron et al. dataset using different prediction models described in Table S1. **Table S12**. Accuracy performance for all PBMC subtypes. Percentile 95% confidence intervals are shown for ten boostrap replicates. **Table S13.** Prediction of dendritic cells from Breton et al. dataset using different prediction models.

**Additional file 3: Table S4.** Prediction results of pancreatic cells without Seurat alignment. **Table S6.** Prediction results using Baron dataset as reference. **Table S7.** Classification performance of scmap-cluster using the Baron dataset as training. **Table S8.** Classification performance of scmap-cell using the Baron dataset as training. **Table S9.** Classification performance of caSTLe using the Baron dataset as training. **Table S10.** Classification performance of singleCellNet using the Baron dataset as training. **Table S11.** Classification performance of scID using the Baron dataset as training. **Table S14**. Differentially expressed genes between unassigned cells by scPred and remaining cord blood-derived cells. **Table S15**. Gene ontology overrepresentation results of overexpressed genes from unassigned cells.

## Data Availability

*scPred* is implemented in R as a package based on S4 objects. The *scPred* class allows the eigen decomposition, feature selection, training, and prediction steps in a straightforward and user-friendly fashion. *scPred* supports any classification method available from the caret package [52]. The default model in *scPred* is the support vector machine with a radial kernel. The choice of this method is based on its superior performance when compared to alternative machine learning methods (Additional file 2: Table S5 and S13). However, it is important to note that the best model will be the one that models the distribution of true effects of the fitted PCs best. Therefore, we anticipate certain scenarios where alternative classification methods should be selected instead of the support vector machine. The *scPred* object contains slots to store the eigen decomposition, informative features selected, and trained models, meaning models can be applied without re-computing the initial training step. The package also includes functions for exploratory data analysis, feature selection, and graphical interpretation. All analyses were run in a personal computer with 16-GB RAM memory and a 2.5-GHz Intel Core i7 processor. *scPred* is available from Github at https://github.com/powellgenomicslab/scPred [57] under the MIT license and on zenodo at doi:10.5281/zenodo.3391594 [58]. Generated data for prediction of tumor cells from gastric cancer may be found in [59]. Data used for prediction of pancreatic cells may be found in GEO (GSE85241, GSE81608, GSE84133) and ArrayExpress (E-MTAB-5061) [60–63]. Data used for prediction of peripheral blood mononuclear cells may be found from 10X Genomics [64]. Data used for prediction of dendritic cells and monocytes may be found in the Single Cell Portal and GEO (GSE89232) [65, 66]. Data used for prediction of colorectal cancer cells may be found in GEO (GSE81861) [67].
