# Supplementary material for: scPred: accurate supervised method for cell-type classification from single-cell RNA-seq data
Source: Genome Biol. 2019 Dec 12;20:264. doi: 10.1186/s13059-019-1862-5 (PMC6907144; doi:10.1186/s13059-019-1862-5)
Supplement: Supplementary file 1 — Additional file 1: Figure S1. Expression of EPCAM, MLH1, and TFF3. Figure S2. Effect of the sequencing depth on prediction performance of gastric tumour cells. Figure S3. Effect of the number of cells on prediction performance of gastric tumour cells. Figure S4. Distribution of conditional class probabilities for single cells from the Baron test dataset across all four models. [file 13059_2019_1862_MOESM1_ESM.pdf]

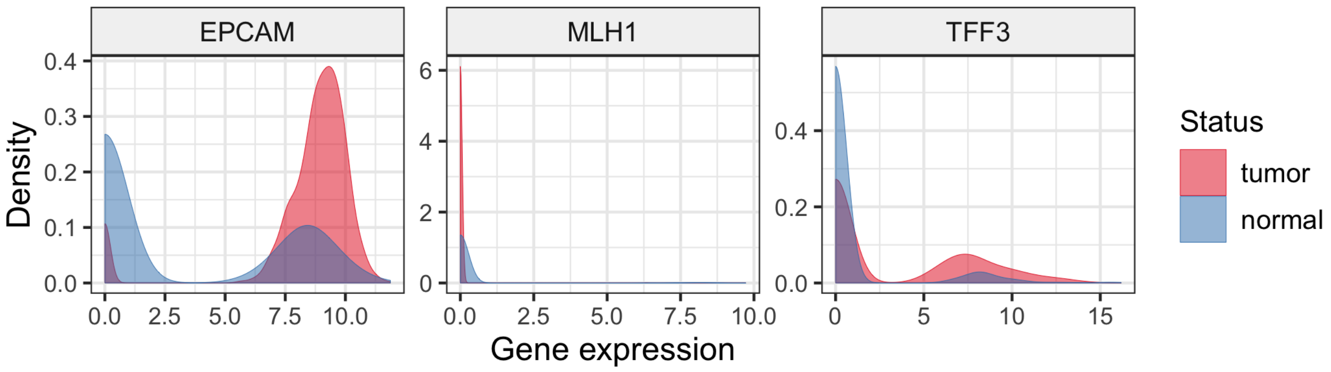

Fig. S1. Expression of EPCAM, MLH1, and TFF3. Expression of EPCAM and TFF3 is upregulated in tumor cells while MLH1 is downregulated. Log2 normalised data is shown for each gene.

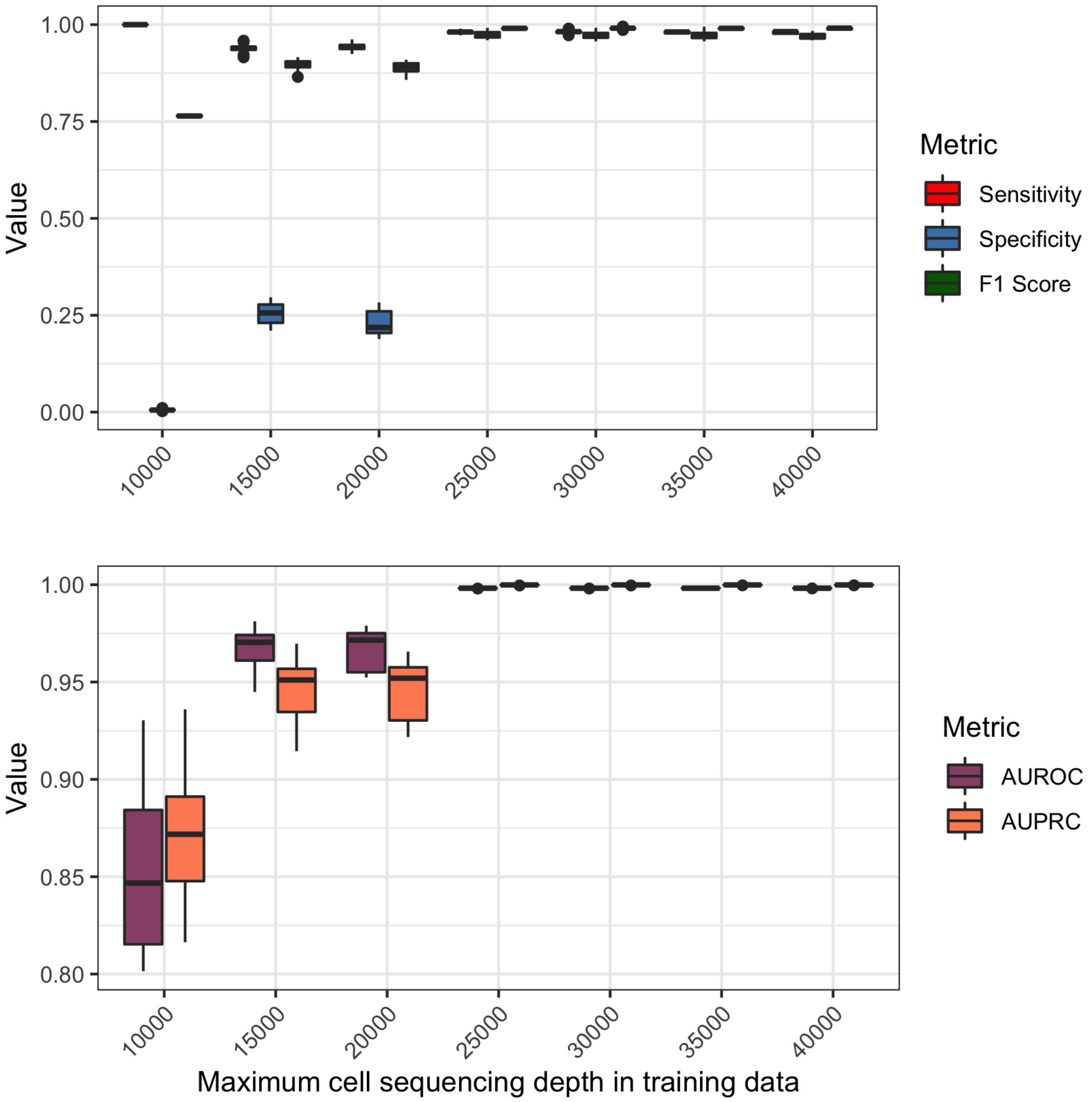

Fig. S2. Effect of the sequencing depth on prediction performance of gastric tumour cells. The sensitivity of the classification showed no changes across sequencing depths, while the specificity, AUROC, and AUPRC show a considerable decrease once the average reads per cell is 20,000. An average sequencing depth of 20,000 reads per cell represents approximately 50% sequencing saturation of the original library. The specificity dropped down to zero when the average sequencing depth was 10,000 reads. Ten bootstrap replicates were performed.

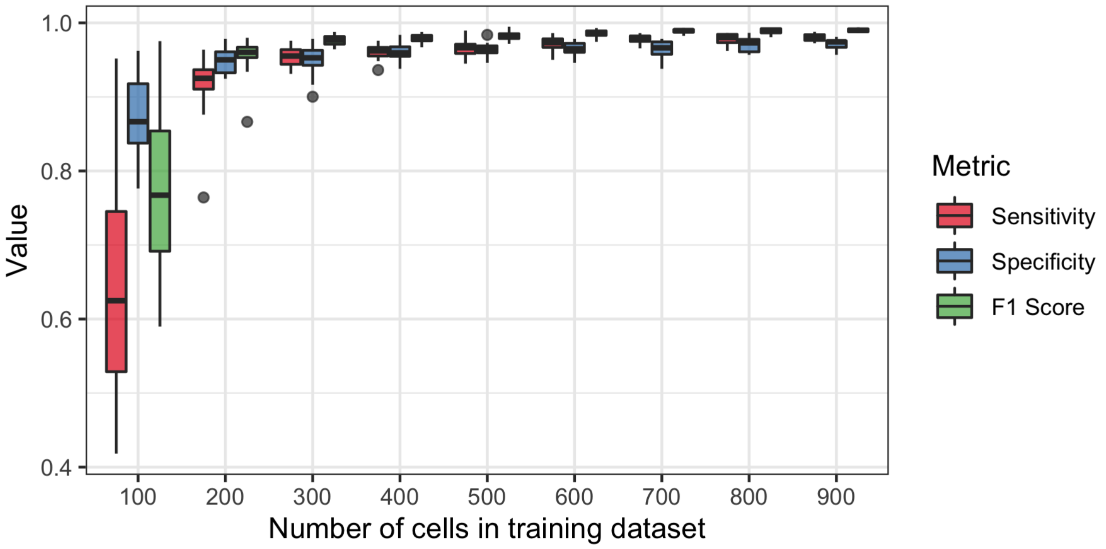

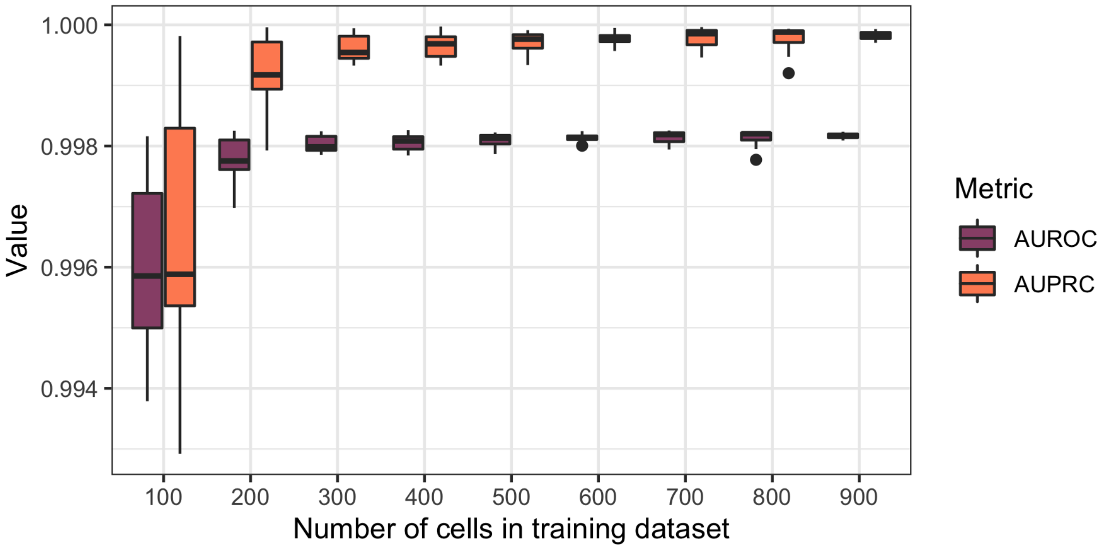

Fig. S3. Effect of the number of cells on prediction performance of gastric tumour cells. Sensitivity and specificity decay proportional directly proportional to the number of cells used to train the classifiers. When only 100 cells were included the mean sensitivity fell to 0.741 whilst the specificity changed from 0.974 to 0.885 and the F1 score from 0.990 to 0.776 with respect to the 953 cells used originally. The AUROC and AUPRC showed minimum decrease. Ten bootstrap replicates were performed.

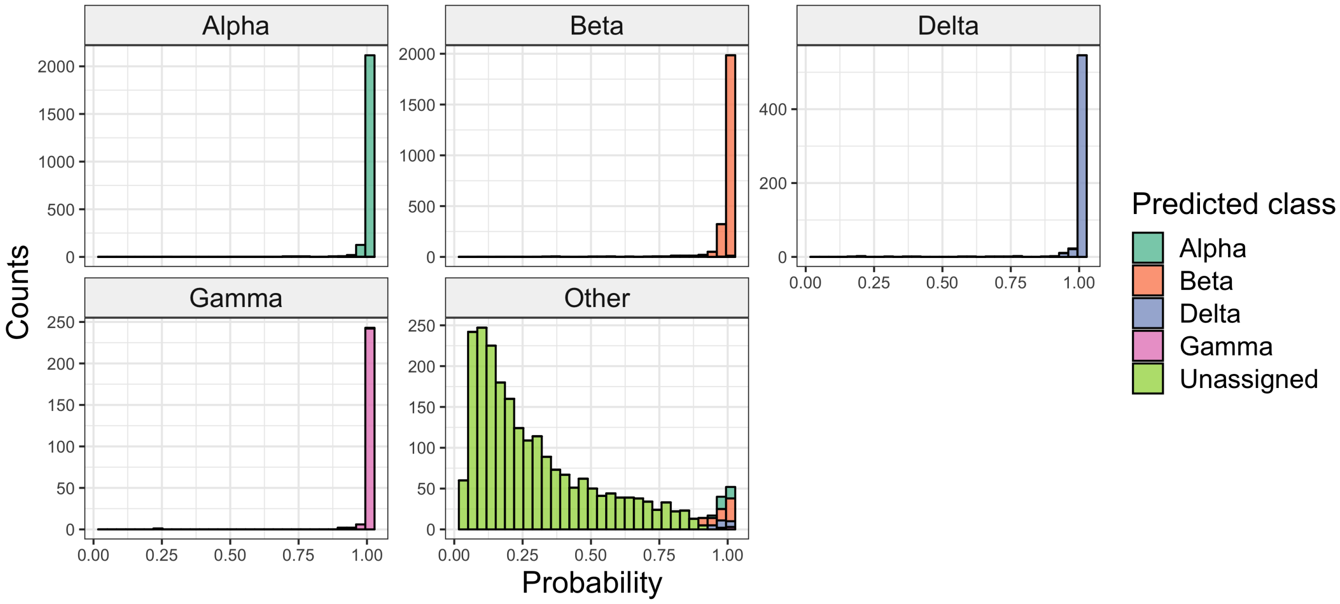

Fig. S4. Distribution of conditional class probabilities for single cells from the Baron test dataset across all four models. Each panel corresponds to the true cell type classes and each color to the predicted class by *scPred*. The right-skewed distributions for α, β, δ, and γ cells indicate a high confidence prediction for most cells from the Islets of Langerhans. The left-skewed distributions for "Other" cells suggests that most of these cells are not likely to belong to any of the cell types of interest.
