# Supplementary material for: scPred: accurate supervised method for cell-type classification from single-cell RNA-seq data
Source: Genome Biol. 2019 Dec 12;20:264. doi: 10.1186/s13059-019-1862-5 (PMC6907144; doi:10.1186/s13059-019-1862-5)
Supplement: Supplementary file 2 — Additional file 2: Table S1. Classification performance comparison between scPred and other prediction baseline methods. For all methods, results are reported using the same data set partitions across all bootstrap replicates. Table S2. Summary of pancreas datasets. Training dataset consisted of 4292 cells and 32 human samples in total. Table S3. Prediction results of pancreatic cells from Baron dataset. Table S5. Prediction performance of pancreatic cells from Baron et al. dataset using different prediction models described in Table S1. Table S12. Accuracy performance for all PBMC subtypes. Percentile 95% confidence intervals are shown for ten boostrap replicates. Table S13. Prediction of dendritic cells from Breton et al. dataset using different prediction models. [file 13059_2019_1862_MOESM2_ESM.pdf]

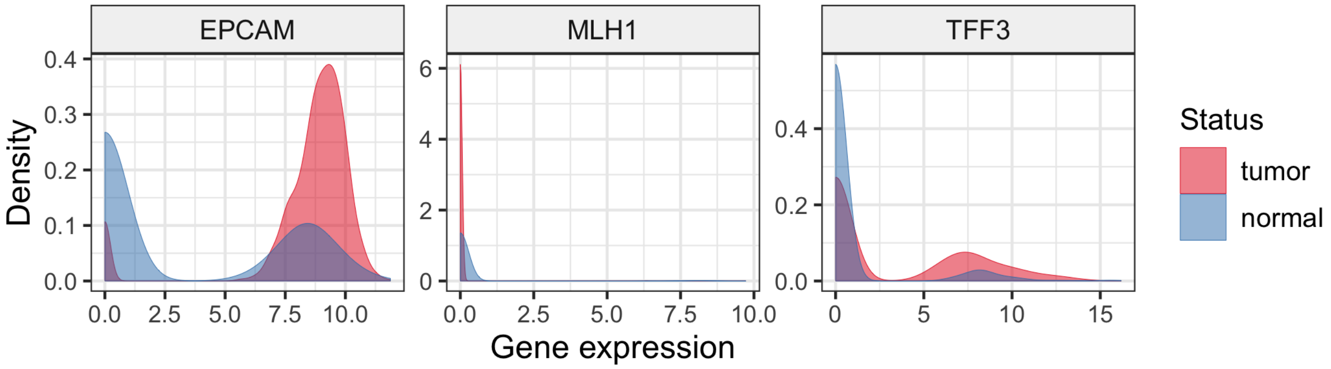

Fig. S1. Expression of EPCAM, MLH1, and TFF3. Expression of EPCAM and TFF3 is upregulated in tumor cells while MLH1 is downregulated. Log2 normalised data is shown for each gene.

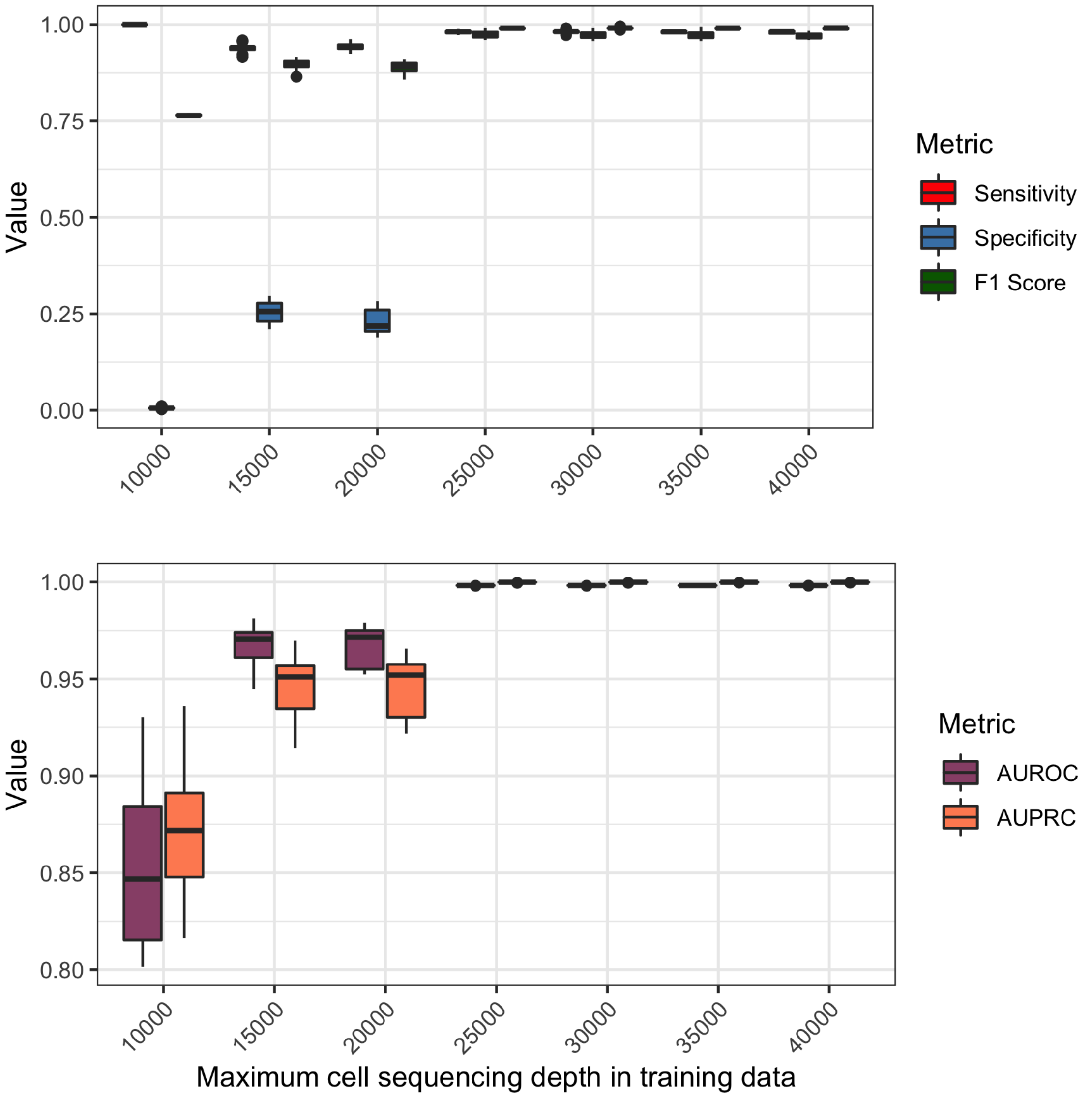

Fig. S2. Effect of the sequencing depth on prediction performance of gastric tumour cells. The sensitivity of the classification showed no changes across sequencing depths, while the specificity, AUROC, and AUPRC show a considerable decrease once the average reads per cell is 20,000. An average sequencing depth of 20,000 reads per cell represents approximately 50% sequencing saturation of the original library. The specificity dropped down to zero when the average sequencing depth was 10,000 reads. Ten bootstrap replicates were performed.

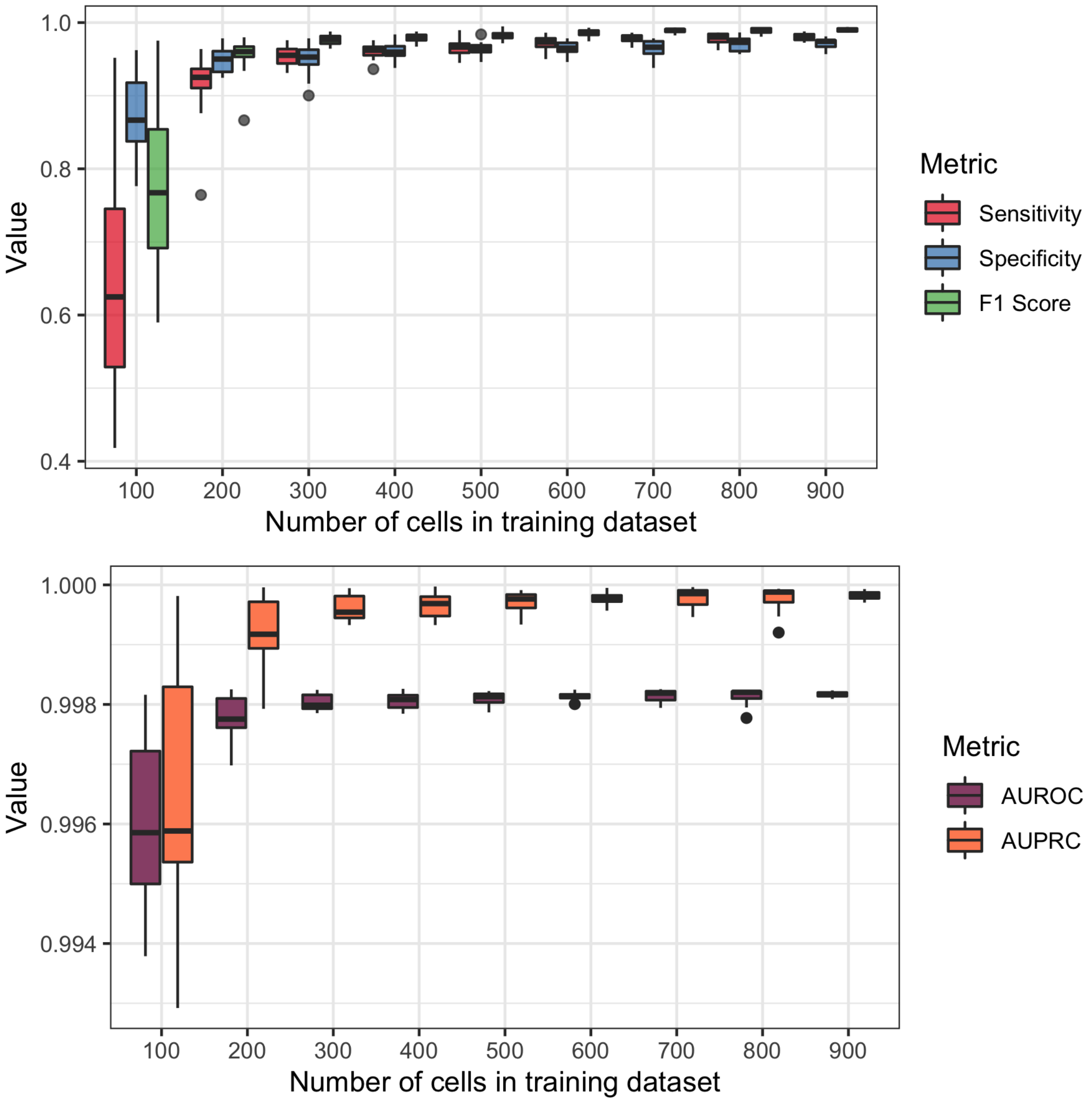

Fig. S3. Effect of the number of cells on prediction performance of gastric tumour cells. Sensitivity and specificity decay proportional directly proportional to the number of cells used to train the classifiers. When only 100 cells were included the mean sensitivity fell to 0.741 whilst the specificity changed from 0.974 to 0.885 and the F1 score from 0.990 to 0.776 with respect to the 953 cells used originally. The AUROC and AUPRC showed minimum decrease. Ten bootstrap replicates were performed.

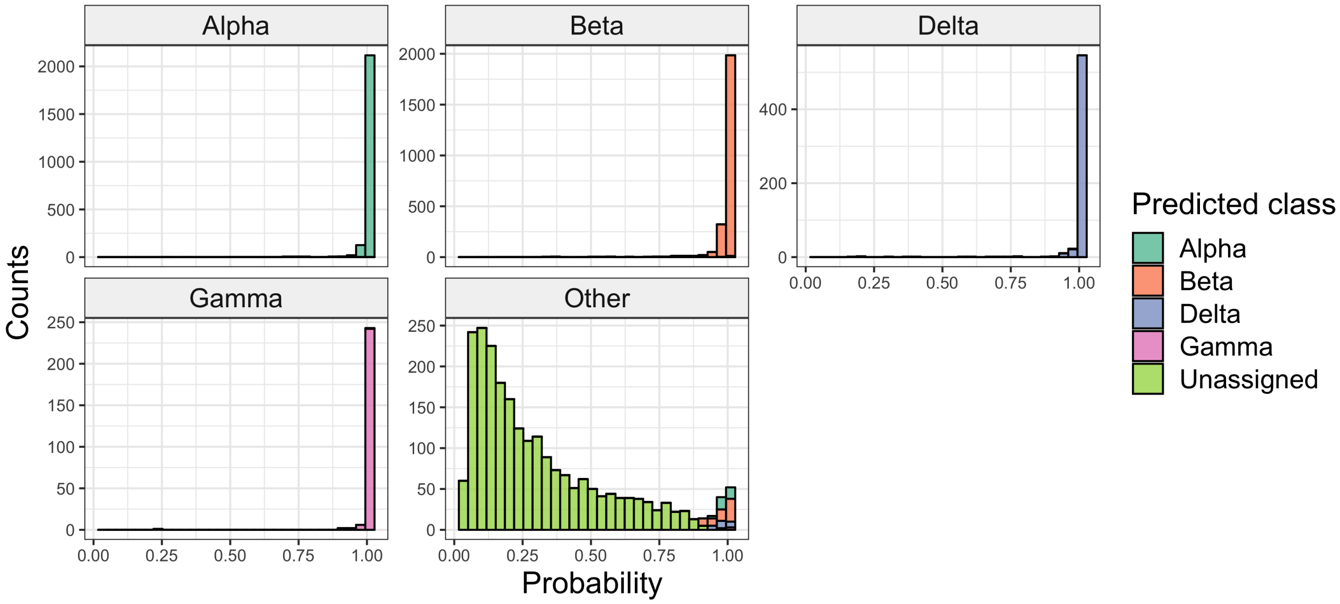

Fig. S4. Distribution of conditional class probabilities for single cells from the Baron test dataset across all four models. Each panel corresponds to the true cell type classes and each color to the predicted class by *scPred*. The right-skewed distributions for α, β, δ, and γ cells indicate a high confidence prediction for most cells from the Islets of Langerhans. The left-skewed distributions for "Other" cells suggests that most of these cells are not likely to belong to any of the cell types of interest.

Method	Sensitivity	Specificity	AUROC	AUPRC	F1 Score
scPred	0.979 ± 0.004	0.974 ± 0.011	1.000 ± 0.000	0.998 ±0.000	0.990 ±0.003
DEGs	0.903 ±0.009	0.909 ±0.014	0.937 ±0.008	0.931 ±0.012	0.922 ±0.010
Coeffs = 1	0.000 ±0.000	0.995 ±0.003	0.496 ±0.003	0.538 ±0.050	0.000 ±0.000
Intercept only	0.000 ±0.000	0.000 ±0.000	0.500 ±0.000	0.000 ±0.000	0.000 ±0.000
All PCs	0.000 ±0.000	0.000 ±0.000	0.000 ±0.000	0.398 ±0.000	0.000 ±0.000

Table S1. Classification performance comparison between scPred and other prediction baseline methods. For all methods, results are reported using the same data set partitions across all bootstrap replicates.

	Dataset	# Cells	# Samples	Protocol	Accession number
Training	Muraro	1522	4	CEL-Seq2	GSE85241
	Segerstolpe	1321	10	Smart-Seq2	E-MTAB-5061
	Xin	1449	18	SMARTer	GSE81608
Testing	Baron	7932	4	inDrop	GSE84133

Table S2. Summary of pancreas datasets. Training dataset consisted of 4,292 cells and 32 human samples in total. All datasets were generated using different protocols. All four samples from the Muraro dataset derive from healthy individuals, as well as 6 samples from the Segerstolpe dataset and 12 from Xin. All the remaining 10 samples from the training reference and 4 from the testing phase come from diabetic individuals. The incorporation of 32 individuals -both healthy and diabetic- to train the prediction model captured a broad biological variability to assess the cell identity of pancreatic cells in other datasets.

Prediction	Alpha	Beta	Delta	Gamma	Other
Alpha	2264	2	0	1	32
Beta	1	2359	0	0	60
Delta	1	13	579	0	21
Gamma	3	2	0	252	5
Lightgray Unassigned	33	78	14	1	2208

Table S3: Prediction results of pancreatic cells from Baron dataset. The first column shows the predicted classes by `scPred` and the remaining columns the true classes. Values along the diagonal corresponds to the number of cells that were correctly classified. The "Unassigned" label is used by `scPred` when a cell cannot be classified with confidence as Alpha, Beta, Delta or Gamma. The "Other" column comprises other cell types except cells from the islets of Langerhans.

Additional file 2: Table S4: Prediction results of pancreatic cells without Seurat alignment. The accuracy for classifying cells from the islets of Langerhans is reduced for all alpha, beta, delta, and gamma cells. Most of the other cell types were classified correctly regardless of the aligment step.

Model	Alpha	Beta	Delta	Gamma	Other
Lightgray SVM Radial	98.3	96.1	97.1	99.2	94.9
k-Nearest Neighbors	84.8	79.5	81.7	86.2	95.4
Elastic net	90.6	90.3	3.9	98.4	95.4
Naive Bayes	93.2	89.4	91.3	97.6	96.1
MARS	96.0	95.5	97.0	97.2	54.6
Random forests	84.5	58.7	11.9	44.1	98.7
GLM	98.2	95.8	97.5	98.4	81.6

Table S5: Prediction performance of pancreatic cells from *Baron et al.* dataset using different prediction models described in table S1. Using a threshold of 0.9 to define class identity for each cell, the support vector machine model with a radial kernel performed better compared to other models as the prediction results show high specificity (for other cells) and high sensitivity (for cell types from the islets of Langerhans).

Additional file 2: Table S6: Prediction results using Baron dataset as reference. Values along the diagonal represent the sensitivities for the target cell-type classes. The average sensitivity and specificity across all three datasets were 0.89 and 0.40 respectively. Contingency tables are also shown for all predictions.

Additional file 2: Table S7: Classification performance of scmap-cluster using the Baron dataset as training. Classifier was applied to Muraro, Segerstolpe, and Xin datasets. All gamma cells were labeled as "unassigned".

Additional file 2: Table S8: Classification performance of scmap-cell using the Baron dataset as training. Classifier was applied to Muraro, Segerstolpe, and Xin datasets. All gamma cells were labeled as "unassigned".

Additional file 2: Table S9: Classification performance of caSTLe using the Baron dataset as training. Classifier was applied to Muraro, Segerstolpe, and Xin datasets. All gamma cells from the Segerstolpe and Xin datasets were labeled incorrectly

Additional file 2: Table S10: Classification performance of singleCellNet using the Baron dataset as training. Classifier was applied to Muraro, Segerstolpe, and Xin datasets. All gamma cells across all datasets had an accuracy lower than 10%

Additional file 2: Table S11: Classification performance of scID using the Baron dataset as training. Classifier was applied to Muraro, Segerstolpe, and Xin datasets. 90% of gamma cells from Muraro dataset were misclassified

Cell type	Mean Accuracy	Bootstrap 95% CI
Blood progenitor	0.9487	0.9452 - 0.9517
Lymphoid	0.9977	0.9973 - 0.9979
Myeloid	0.9638	0.9550 - 0.9777
B cells	0.9975	0.9980 - 0.9996
Natural killer	0.9865	0.9902 - 0.9940
T cells	0.9949	0.9955 - 0.9984
Cytotoxic T cells	0.9513	0.9543 - 0.9617
Non-cytotoxic T cells	0.9739	0.9764 - 0.9796

Table S12: Accuracy performance for all PBMC subtypes. Percentile 95% confidence intervals are shown for ten boostrap replicates.

Model	Peripheral blood accuracy	Cord Blood accuracy
Lightgray SVM Radial	97.8	70.2
k-Nearest Neighbors	96.1	76.9
Elastic net	90.7	61.7
Naive Bayes	96.3	67.1
MARS	89.8	62.1
Random forests	45.1	36.4
GLM	85.2	58.6

Table S13: Prediction of dendritic cells from *Breton et al.* dataset using different prediction models. Except from random forests, all models showed a high accuracy for dendritic cells from peripheral blood. For cord blood-derived cells, wide differences are observable across models due to the presence of other subpopulations. The support vector machine model showed the best accuracy for peripheral blood-derived dendritic cells. Accuracy is defined as the fraction of real dendritic cells correctly predicted by *scPred*.

Additional file 2: Table S14: Differentially expressed genes between unassigned cells by scPred and remaining cord blood-derived cells. Top upregulated genes include T-cell receptor gamma locus and myeloid-related genes.

Additional file 2: Table S15: Gene ontology overrepresentation results of overexpressed genes from unassigned cells. A Fisher's exact with FDR multiple test correction. Biological processes involving myeloid and neuthophiles were overrepresented. X06776, XIST, BC039116, M64936, TARP, FCGR1C, and ECRP gene identifiers did not map the query database.
